# The genome of single-petal jasmine (*Jasminum sambac*) provides insights into heat stress tolerance and aroma compound biosynthesis

**DOI:** 10.3389/fpls.2022.1045194

**Published:** 2022-10-19

**Authors:** Xiangyu Qi, Huadi Wang, Shuangshuang Chen, Jing Feng, Huijie Chen, Ziyi Qin, Ikram Blilou, Yanming Deng

**Affiliations:** ^1^ Jiangsu Key Laboratory for Horticultural Crop Genetic Improvement, Institute of Leisure Agriculture, Jiangsu Academy of Agricultural Sciences, Nanjing, China; ^2^ School of Life Sciences, Jiangsu University, Zhenjiang, China; ^3^ College of Horticulture, Nanjing Agricultural University, Nanjing, China; ^4^ Biological and Environmental Sciences and Engineering, King Abdullah University of Science and Technology, Thuwal, Saudi Arabia

**Keywords:** genome evolution, heat stress, benzenoid/phenylpropanoid biosynthesis, terpenoid biosynthesis, terpene synthase

## Abstract

Jasmine [*Jasminum sambac* (L.) Aiton] is a commercially important cultivated plant species known for its fragrant flowers used in the perfume industry, medicine and cosmetics. In the present study, we obtained a draft genome for the *J. sambac* cultivar ‘Danbanmoli’ (JSDB, a single-petal phenotype). We showed that the final genome of *J. sambac* was 520.80 Mb in size (contig N50 = 145.43 kb; scaffold N50 = 145.53 kb) and comprised 35,363 genes. Our analyses revealed that the *J. sambac* genome has undergone only an ancient whole-genome duplication (WGD) event. We estimated that the lineage that has given rise to *J. sambac* diverged from the lineage leading to *Osmanthus fragrans* and *Olea europaea* approximately 31.1 million years ago (Mya). On the basis of a combination of genomic and transcriptomic analyses, we identified 92 transcription factors (TFs) and 206 genes related to heat stress response. Base on a combination of genomic, transcriptomic and metabolomic analyses, a range of aroma compounds and genes involved in the benzenoid/phenylpropanoid and terpenoid biosynthesis pathways were identified. In the newly assembled *J. sambac* genome, we identified a total of 122 MYB, 122 bHLH and 69 WRKY genes. Our assembled *J. sambac* JSDB genome provides fundamental knowledge to study the molecular mechanism of heat stress tolerance, and improve jasmine flowers and dissect its fragrance.

## Introduction

Jasmine [*Jasminum samba*c (L.) Aiton] is a diploid (2n = 2x = 26) evergreen ornamental species belonging to the family Oleaceae. It is one of the most important commercial flower plant species in many countries, and be used extensively in bouquets, ornamental displays, tea, cosmetics and perfumery (Cai et al., 2007; Wikee et al., 2011). In China, jasmine cultivation dates back more than 2,000 years on account of its usage in traditional Chinese medicine and its high value in scenting the famous ‘jasmine tea’ (Deng et al., 2016). Jasmine plants generally exhibit single-, double-, or multi-petal phenotypes (Deng et al., 2017). Among which the *J. sambac* cultivar ‘Danbanmoli’ (JSDB, a single-petal phenotype) is one of the main cultivars cropped in China, for its flowers are considered to be the most fragrant (Deng et al., 2017). The perfumed flowers contain an essential oil known as the “attar of jasmine”, which is rich in low molecular weight aroma compounds, and the most prominent of which are benzenoids, phenylpropanoids and terpenoids (Bera et al., 2017).

In plants, benzenoids/phenylpropanoids are generated *via* the aromatic acid phenylalanine produced from phenylpyruvate and arogenate pathways, which diverge from the shikimate pathway (Maeda and Dudareva, 2012; Qian et al., 2019). Monoterpenes/diterpenes and sesquiterpenes are derived from the 2-C-methylerythritol-4-phosphate (MEP) and mevalonate (MVA) pathways, respectively (Vranová et al., 2013). Studies have identified numerous genes involved in the phenylpropanoid, MEP and MVA pathways (Achnine et al., 2004; Degenhardt et al., 2009; Olofsson et al., 2011). Furthermore, transcription factors (TFs), including MYB, bHLH and WRKY families are also involved in terpenoids synthesis (Shang et al., 2020). However, the molecular mechanism of aroma compounds biosynthesis in jasmine is still not very clear and needs further exploration.

As global warming progresses, heat stress is becoming a threat to the environment as well as plant populations (Parmesan and Yohe, 2003). Heat stress compromises plant growth by causing reactive oxygen species generation, protein denaturation and membrane destabilization (Mittler et al., 2012). Under heat stress, heat shock transcription factors (HSFs) are rapidly activated and enhance the expression of many genes that encode heat shock proteins (HSPs) (Ohama et al., 2016). HSFs are core regulators of the heat stress response. HSPs protect cellar components by preventing protein denaturation and aggregation. *J. sambac* is one of the evergreen species that bloom in the hot summer, during which the temperature rises above 38°C. Therefore, it is important to elucidate the molecular mechanism involved in the heat stress tolerance of jasmine, and this will be helpful to understand the plant’s adaptability to high temperature condition.

Genomic resources are essential for molecular and evolutionary studies and are becoming increasingly attainable. With the ongoing rapid developments in sequencing technology, an increasing number of genomes are being sequenced and released (Chen et al., 2018a; Chen et al., 2019). To date, several species in the Oleoideae subfamily of the Oleaceae, including *Fraxinus exceisior* (Sollars et al., 2017), *Olea europaea* (Unver et al., 2017), *Osmanthus fragrans* (Yang et al., 2018), and *Forsythia suspensa* (Li et al., 2020) have been sequenced and reported. Jasmine cultivars are characterized by differing floral phenotypes ranging from single- to multi-petal flowers. These differences not only have a notable influence on floral morphology, but also influence floral fragrance, because petals contribute to producing aroma components. Although a genome of *J. sambac* cultivar ‘Trifoliatum’ was reported recently (Xu et al., 2021), there is currently a lack of genomic resource for single-petal jasmine, which is essential for linking an understanding of flower fragrance biosynthetic genes with aroma scent emission during flowering.

In this study, we obtained a draft genome of *J. sambac* single-petal cultivar JSDB based on PacBio and Illumina sequencing. On the basis of a combination of genomic and transcriptomic analyses, we identified TFs and genes related to its heat stress response. Base on a combination of genomic, transcriptomic, and metabolomic analyses, we identified a range of aroma compounds and genes involved in the benzenoid/phenylpropanoid and terpenoid biosynthesis pathways. Our data provide new insights into the molecular mechanisms underlying heat stress tolerance and aroma scent emission of *J. sambac*.

## Materials and methods

### Plant materials, library construction and sequencing

The *J. sambac* JSDB was maintained in the Preservation Centre of the Jasmine Germplasm Resources, Jiangsu Academy of Agricultural Sciences, Nanjing, China (latitude: 32°05′N, longitude: 118°08′E; 68 m above sea level) (**Figure S1**). Tender young leaves of individual plant were collected from *J. sambac*. The genomic DNA was extracted using the cetyltrimethylammonium bromide (CTAB) method (Li et al., 2013). A total of four PacBio 20-kb libraries were generated using an SMRTbell Template Prep Kit (PacBio) and were sequenced on the PacBio Sequel platform (**Table S1**). For Illumina library construction, the genomic DNA was fragmented and size-fractionated, then subjected to library construction and sequenced on the Illumina HiSeq 2000 system with paired-end 150-bp reads (**Table S1**). All the above sequencing was performed by Shanghai Personal Biotechnology Company Limited (Shanghai, China).

### Estimation of *J. sambac* genome size

Fresh leaves were collected from the same *J. sambac* plant used for sequencing. The genome size was determined based on flow cytometry (Doležel et al., 2007), with three parallel experiments being carried out for each sample. The *Solanum lycopersicum* L. ‘Stupicke polni tyckove rane’ was used as an internal standard. The sequencing data were analyzed through FACSTM 1.0.0.650 software, and the statistical analysis was conducted using SPSS 17.0.

The *J. sambac* genome size was also estimated by using a *k*-mer (*k* = 17) analysis-based approach with quality-filtered Illumina paired-end short reads. Jellyfish software (version 2.1.4) was applied for counting *k*-mers in the DNA samples (Marcais and Kingsford, 2011). GCE software (version 1.0) was used for estimating genome size (Liu et al., 2013). Finally, the heterozygosity of JSDB was determined (Kajitani et al., 2014).

### Genome assembly and quality assessment

Raw Illumina reads were processed to collapse duplicated read pairs into unique read pairs. Duplicated read pairs were defined as those having identical bases at positions 14 to 90 in both left and right reads. Then, the resulting reads were processed to remove adaptor and low-quality sequences using AdapterRemoval (version 2.1.7) (Schubert et al., 2016). Reads shorter than 50 bp at either end was further discarded. Finally, sequencing errors in paired-end reads were corrected using SOAPec (parameter ‘-kmer-len 17’) (Luo et al., 2012).

For *J. sambac* genome assembly, we used DBG2OLC (Ye et al., 2016). The high-quality cleaned paired-end reads were initially assembled into contigs using Platanus (version 1.8.8 parameters ‘-k 32 -s 10 -c 2 -a 10.0 -u 0.1 -d 0.5’), and thereafter connected to scaffolds with DBG2LOC using all paired-end reads. Following assembly, the third-generation sequencing raw data were used to correct the scaffolds using Arrow (version 2.2.2) in two rounds, and the high-quality next-generation sequencing data were then used to correct scaffolds in a further two rounds using Pilon (version 1.22) with paired-end reads. To evaluate the accuracy and completeness of the genome assemblies, BUSCO (version 3.0.2) was performed using the embryophyta_odb10 plant database (Simão et al., 2015).

### Repetitive elements and non-coding RNA annotation

In order to search for transposable elements in the assembled *J. sambac* genome, an integrated strategy based on *de novo* prediction and a homology-based method was adopted. For *de novo* prediction, we identified repetitive elements using RepeatModeler (version 1.0.4; http://www.repeatmasker.org/RepeatModeler/), RECON (version 1.0.8; http://selab.janelia.org/recon.html) and RepeatScout (version1.0.5; http://repeatscout.bioprojects.org/), with default parameters. Homology-based repetitive elements were identified by comparison with consensus sequences in the Repbase library (version 20150807) using RepeatMasker (version 4.0.5; http://www.repeatmasker.Org/) (Kapitonov and Jurka, 2008).

We searched for LTR-RTs in the genome using LTR_finder with parameters ‘-D 5000 -d 100 -L 20000 -l 1000 -p 20 -M 0.3’ (Xu and Wang, 2007) and LTRharvest with parameters ‘-v -mintsd 4 -maxtsd 6’ (Ellinghaus et al., 2008). Then, the identified LTR-RT candidates were filtered using LTRdigest (Steinbiss et al., 2009) with default parameters ‘-trnas -hmms’. The insert time (T) of intact LTRs was estimated using the formula T = K/2r, where K is the number of nucleotide substitutions per site between each pair of LTRs and r refers to the general nucleotide substitution rate, which was set to 1.3 × 10^-8^ per site per year (Ma et al., 2020).

We predicted tRNAs using tRNAscan-SE (version 1.3.1) (Lowe and Eddy, 1997). rRNAs were predicted using RNAmmer (version 1.2) (Lagesen et al., 2007), and other ncRNAs were predicted using the Perl program Rfam_-_scan.pl (version 1.0.4) by inner calling using Infernal (version 1.1.1) (Nawrocki and Eddy, 2013).

### Gene prediction and annotation

Based on the repeat-masked genome, we combined evidence obtained from three source (ab *initio* gene prediction, homolog searching and UniGene-based prediction) to predict non-redundant protein-encoding gene models. For *ab initio* gene prediction, Augustus (version 3.0.3) (Stanke and Morgenstern, 2005), SNAP (version 2006-07-28) (Korf, 2004), and GlimmHMM (version 3.0.4) (Majoros et al., 2004) were used to annotate genes, whereas, for the homolog-based prediction, we mapped the *J. sambac* genome against the published protein sequences of *A. thaliana*, *Erythranthe guttata*, *O. europaea*, *Sesamum indicum* and *Vitis vinifera* using Exonerate (version2.2.0, http://www.animalgenome.org/bioinfo/resources/manuals/exonerate/exonerate.man.html). To accurately identify alignments, we used GeneWise (version 2.4.1) to filter the initially aligned coding sequences (Birney et al., 2004), and for the UniGene-based prediction, Trinity (version r20140717) was used to assemble the RNA-seq data (Haas et al., 2013). Thereafter, we applied PASA software (version r20140417) to improve the gene structure (Haas et al., 2008). All three prediction methods were then integrated by EvidenceModeler (version r2012-06-25) (Haas et al., 2008). Finally, we used PASA software (Haas et al., 2008) to obtain annotation information for the 5′ and 3′ UTRs of genes, as well as variations in alternative splicing.

We performed functional annotation of the genes based on BLASTP (E-value< e^-6^) searches against the NCBI NR, SwissProt and eggNOG (version 4) databases (Shang et al., 2020). We determined the motifs and domains of genes using InterProScan (version 5.28) (Jones et al., 2014), whereas we determined the Gene Ontology (GO) classification of genes using InterPro or Pfam entry, and obtained KO and Pathway annotations of protein-coding genes using KAAS (Moriya et al., 2007) and the KEGG database.

### Genome comparison and phylogenomic analysis

To identify orthologous genes among 15 plant genomes, the complete genome sequences of 14 other plants (*A. thaliana*, *Begonia fuchsioides*, *Beta vulgaris*, *Betula pendula*, *Cercis canadensis*, *Durio zibethinus*, *Helianthus annuus*, *Nelumbo nucifera*, *Oryza sativa*, *Prunus mume*, *Solanum pennellii*, *V. vinifera*, *O. europaea* and *O. fragrans*) from the appropriate websites were retrieved (**Table S2**). We used OrthoFinder (version 2.2.6) pipeline to identify gene families (Emms and Kelly, 2019), and then single-copy orthologs genes were used for MUSCLE alignment. We constructed phylogenomic tree using RAxML (version 8.2.12) (Stamatakis, 2014). We estimated divergence times among the 15 examined plant species using the program Mcmctree (version 4.0) in the PAML package (version 4.8) (Yang, 2007), with three corrected divergence times point [*A. thaliana*-*O. sativa* (148–173 Mya), *A. thaliana*-*V. vinifera* (105–115 Mya) and *A. thaliana*-*D. zibethinus* (81–94 Mya) obtained from the TimeTree website (http://www.timetree.org/)] being used to adjust the divergence times. Expansion and contraction events in gene families were computationally identified using cafe` (version 3.0) software (De Bie et al., 2006).

### Whole-genome duplication analysis

We used four-fold synonymous third-codon transversion (4DTv) to estimate whole-genome duplication (WGD) events. Initially, the respective paralogs of *J. sambac*, *V. vinifera*, *O. europaea* and *A. thaliana*, and the respective orthologs of *J. sambac* and *V. vinifera*, *J. sambac* and *O. europaea* and *J. sambac* and *A. thaliana* were identified. Then, we identified the conserved paralogs and orthologs based on BLASTP (E-value< e^-5^) searches, and calculated WGD events based on their 4DTv values.

### Aroma compounds analysis

Fresh flowers at three different stages of development (S1, young floral bud stage; S2, mature floral bud stage; S3, initial opening flower stage), defined by flower size, were picked from the same plants at the time of samples collection for transcriptome study (**Figure S2**). To identify aroma compounds and determine the quantity of floral scent, headspace solid-phase microextraction (HS-SPME) combined with gas chromatography-mass spectrometry (GC-MS) was used (Yang et al., 2018). The spectra obtained for volatile compounds were auto-matched based on comparisons with those in the NIST08 mass spectral library and those reported in specialized literature. The quantities of the volatile compounds were based on the normalization of peak areas.

### Transcriptome libraries preparation and sequencing

To obtain information that can be used to assist gene annotation, we collected mixed tissues (leaves, stems, roots, buds and flowers at the aforementioned three developmental stages) from the *J. sambac* plant used for genome sequencing. We also collected flowers at the three stages from three plants at the time of sample collection for identifying aroma compounds (each with three biological replicates) (**Figure S2**). We isolated total RNA using Trizol Reagent (Invitrogen Life Technologies, USA). The concentration, quality and integrity of which were determined using a NanoDrop spectrophotometer (Thermo Fisher Scientific, USA). We generated sequencing libraries using a TruSeq RNA Sample Preparation Kit (Illumina, San Diego, CA, USA), and these were subsequently sequenced with the HiSeq 2000 platform using paired-end sequencing with 150-bp reads (**Table S1**). We mapped the RNA-seq reads of each sample to the reference genome of *J. sambac*.

### Identification and analysis of heat stress response and aroma compound biosynthesis genes

Using heat stress response related genes from *A. thaliana* (Ohama et al., 2017), terpene biosynthesis genes from *A. thaliana* (Vranová et al., 2013) and phenylpropanoid biosynthesis genes from *Petunia hybrida* (Maeda and Dudareva, 2012) as baits, the corresponding genes in *J. sambac* were identified based on genome annotation and local blast searches against *J. sambac* genome using a filtered parameter (E-value < 10^−6^, identity ≥ 40% and coverage ≥ 30%). *TPS* genes in the *J. sambac* genome were identified as previously reported (Song et al., 2018). Multiple sequence alignment of the amino acid sequences of *TPS* genes, *Hsfs*, MYB TFs, bHLH TFs, and WRKY TFs were performed using the default parameters of MUSCLE followed by maximum likelihood phylogenetic analyses performed using RAxML (version 8.2.12) (Stamatakis, 2014). The 3000-bp upstream region of *TPS* genes was defined as the promoter fragment, and the *cis*-acting regulatory elements within these promoter sequences were identified using PlantCARE (Lescot et al., 2002) and PLACE (Higo et al., 1999). Based on the expression levels of genes at the three different floral developmental stages, a heat map was illustrated using TBtools (Chen et al., 2018b).

## Results

### Determination of genome size and heterozygosity

Given genome size varies between species and cultivars, using flow cytometry, we sought to determine the *J. sambac* genome size using fresh leaves from the same individual used for genome sequencing (**Figures S3A**), and accordingly established that the size of the single-petal *J. sambac* genome was 583 Mb (**Table S3**).

The *J. sambac* genome size and the rate of heterozygosity were also estimated from raw short-reads sequenced using the *k*-mer based method. A total of 54.67 Gb short paired-end reads (2 × 150 bp) was obtained by Illumina sequencing, of which 52.36 Gb remained after filtering out low-quality reads (**Tables S1**, **S4**). The 17-mer frequency of short reads with the main peak appeared at a depth of 84 (**Figure S3B**). On the basis of these data, the genome size was estimated to be 555.45 Mb, and the rate heterozygosity was 0.84% (**Table S5**).

### Sequencing and assembly of the *J. sambac* genome

For the *J. sambac* genome, we generated 54.7 Gb (~98×) Illumina and 15.9 Gb (~28×) PacBio reads (**Tables S4**, **S6**). The integrated work-flow of the genome assembly was shown in **Figure S4**. The assembly yielded a draft genome of 521 Mb, representing ~94% (521 Mb/555 Mb) of the estimated genome size, with contig N50 and scaffold N50 length values of 145.43 and 145.53 kb, respectively (**Table 1**) and 95.0% BUSCO completion (**Table S7**).

**Table 1:** Statistics of *J. sambac* cultivar JSDB genome assembly.

Property	Contig	Scaffold
Total sequence number	6,916	6,904
Total sequence length (bp)	520,804,524	520,804,524
GC content (%)	34.43%	34.43%
Sequences greater than 1kb	6,916	6,904
Shortest (bp)	1,206	1,206
Longest (bp)	1,705,941	1,705,941
N20 (bp)	311,297	312,206
N50 (bp)	145,430	145,534
N90 (bp)	30,874	30,964

### Annotation of the genome

On the basis of a combination of *de novo* annotation and homolog-based approaches, 49.01% of the *J. sambac* assembled genome was identified as being repetitive sequences (**Table 2**). It was found that most of these replicated sequences were transposable elements (TEs) (comprising 48.64% of the genome) (**Table 2**). Among the major types of TEs identified, long terminal repeat retrotransposons (LTR-RTs) comprised the largest proportion (accounting for 20.56% of the genome). In the genome, 11.23% of LTRs were *Gypsy* elements and 9.20% were *Copia* elements (**Table 2**). Unclassified repeats ranked the second most abundant, accounting for 20.24% of the genome (**Table 2**). In addition to the LTRs and unclassified repeats, 6.14% of the genome were annotated as DNA transposons, and 1.70% as long interspersed nuclear elements (LINEs), with the remaining repeats being assigned to other elements (**Table 2**). Moreover, 3,394 complete LTR-RTs were identified in the genome, and the estimated time of LTR-RT burst was approximately 0.2 million years ago (Mya) (**Figure S5**). Non-coding RNA (ncRNA) genes were also annotated, and accordingly we identified 261 miRNAs, 630 tRNAs, 122 rRNAs and 781 snRNAs, respectively (**Table S8**).

**Table 2:** Statistics of repetitive element contents in the *J. sambac* cultivar JSDB genome.

Elements	Number	Length (bp)	Percentage of Genome
TE		253,299,380	48.64%
LTR	94,608	107,064,411	20.56%
LTR/Copia	42,421	47,920,897	9.20%
LTR/Gypsy	62,747	58,472,652	11.23%
LINE	16,379	8,827,688	1.70%
SINE	273	26,269	0.01%
DNA	95,358	31,985,436	6.14%
Satellite	2,675	740,381	0.14%
Simple repeat	5,305	2,233,154	0.43%
Low complexity	11	1969	0.00%
Unclassified	335,444	105,395,576	20.24%
Total	550,053	255,240,103	49.01%

For the purposes of identifying protein-coding genes, a combination of homolog-based prediction, ab *initio* prediction and transcriptome-assisted prediction were used, which enabled us to predict final set of 35,363 protein-coding genes, with an average transcript length of 3,323.1 bp, an average coding sequence length of 1,025.3 bp, and an average of 4.8 exons per gene (**Table S9**). Among the annotated genes, 29,921 (84.6%) of the genes were functionally classified based on reference to five databases (**Table S10**).

### Genome evolution

To investigate the evolutionary position and distinct traits of single-petal jasmine, a comparative analysis of the genome of *J. sambac* and 14 other plant species was performed (**Table S2**). On the basis of the proteomic databases, we identified 61 gene families unique to *J. sambac*, comprising 519 genes (**Figure S6**, **Table S11**).

**Figure 1:**
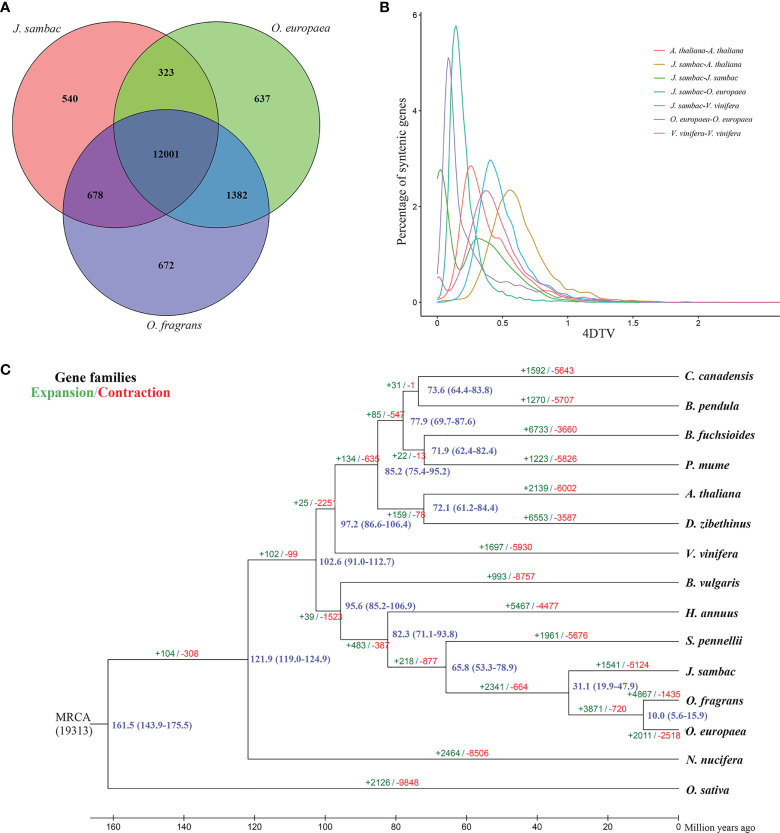
Evolution and comparative analysis of the *J*. *sambac* cultivar JSDB genome. **(A)** Venn diagram of *J*. *sambac*, *O. europaea* and *O. fragrans*. Each number in the diagram was the gene family number within a group. **(B)** Fourfold degenerate distributions for *J*. *sambac*, *O. europaea*, *A*. *thaliana*, and *V. vinifera*. **(C)** Phylogenomics relationships, divergence times and gene family expansion and contraction of 15 plant species. The blue numbers on the nodes were divergence time to present (in Mya). The green and red numbers above or under each branch denoted the expanded and contracted gene families after the diversification from the most recent common ancestor (MRCA), respectively.

To evaluate the phylogenetic relationships between *J. sambac* and other plant species, a phylogenomic tree based on 352 single-copy genes was constructed, and thereby we estimated the divergence times. It revealed that *J. sambac* was closely related to the fragrant tree (*O. fragrans*) and the European olive (*O. europaea* ***)*** (**Figure 1C**). The lineage giving rise to *J. sambac* was found to diverge from that leading to *O. fragrans* and *O*. *europaea* at ~31.1 Mya, and the lineage that gave rise to the Oleaceae species diverged from the lineage giving rise to *S. pennellii* at ~65.8 Mya (**Figure 1C**). Moreover, 1,541 gene families were found to have undergone an expansion, whereas 5,124 gene families have undergone contractions (**Figure 1C**).

A comparison of the genomes of *J. sambac*, *O. europaea* and *O. fragrans* revealed that 12,001 gene families were common to these Oleaceae species, whereas 540 gene families were identified as being unique to *J. sambac* genome (**Figure 1A**). We further applied 4DTv analysis to investigate *J. sambac* WGD events, and the analysis indicated that *J. sambac* has undergone only an ancient WGD event (**Figure 1B**).

### Genes involved in heat stress

**Figure 2:**
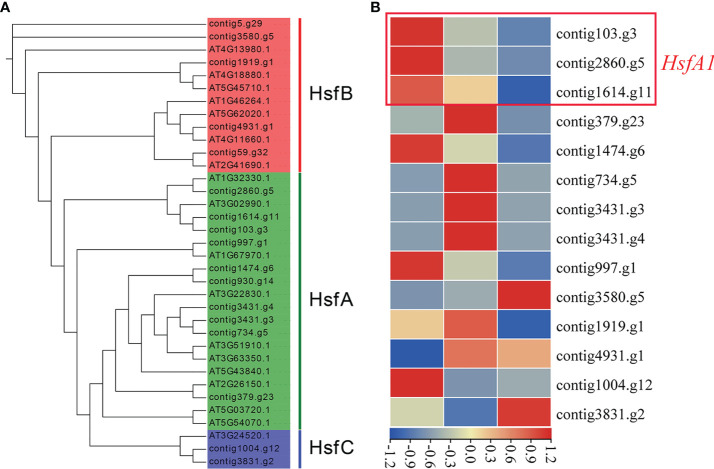
Phylogenetic analysis of *Hsf* transcriptional factors identified in the *J*. *sambac* cultivar JSDB and *A*. *thaliana* genomes **(A)** and the expression of *Hsfs* in the three floral developmental stages of *J*. *sambac* cultivar JSDB **(B)**. S1, young floral bud stage; S2, mature floral bud stage; S3, initial opening flower stage.

In order to reveal the heat stress tolerance mechanism of *J. sambac*, we identified genes related to heat stress response, and accordingly 92 transcription factors (TFs) and 206 genes were identified (**Tables S12–S14**). We found a set of genes had expanded in both *J. sambac* and *H. annuus* compared with *A. thaliana*, including dehydration-responsive element-binding protein 2 (*DREB2*), *NAC* (NAM, ATAF and CUC), squamosa-promoter binding-like (*SPLs*), heat shock proteins (*HSP*), nuclear factor Y subunit A2 (*NF-YA2*), nuclear factor Y subunit B3 (*NF-YB3*), cyclin-dependent kinase A1 (*CDKA1*), calmodulin-binding protein kinase 3 (*CBK3*), heat-intolerant 4 (*HIT4*), decrease in DNA methylation 1 (*DDM1*) and cyclic nucleotide-gated channel (*CNGCs*) (**Tables S12**, **S13**). Furthermore, three *HsfA1s* that act as the master regulators in the heat response were identified in the 17 *Hsf* genes (**Figure 2A**; **Table S14**). *HsfA1s* and *DREB2A* displayed the same expression pattern, and both of them had high expression levels at the S1 stage. Meanwhile, the abundance of their transcript decreased with the flower development (**Figures 2B**; **Figure S7**).

### Genes involved in benzenoid/phenylpropanoid biosynthesis

To establish a direct linkage between biosynthetic genes and flower fragrance development, a metabolomic approach was used to determine the aroma compounds synthesized at three flower developmental stages (**Figure S2**). On the basis of HS-SPME/GC-MS combined analysis, we accordingly identified over 50 aroma compounds (**Figure S8**; **Table S15**).

**Figure 3:**
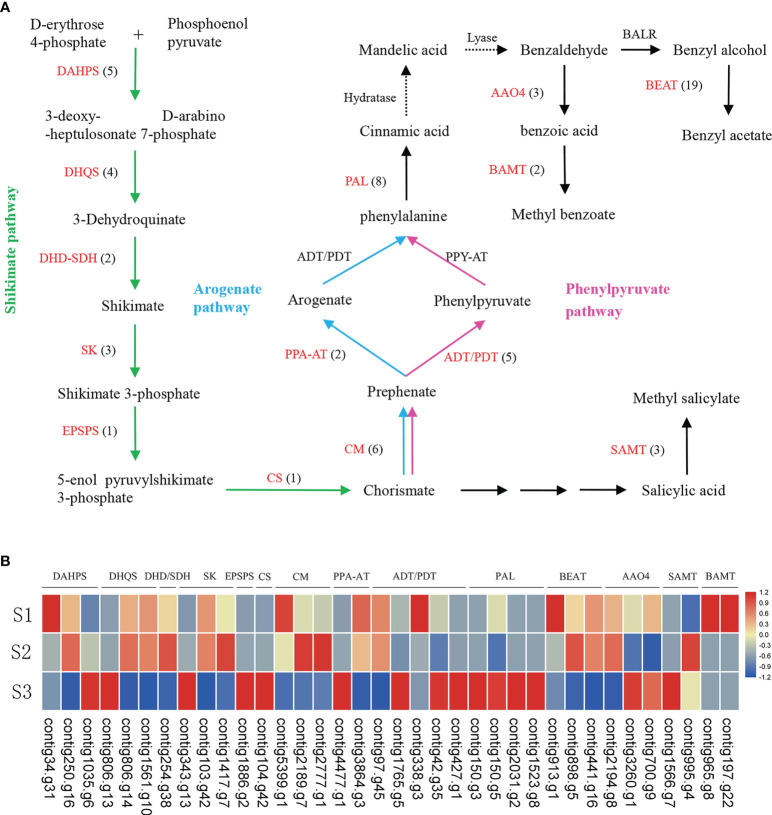
The biosynthesis pathways of benzenoid/phenylpropanoid **(A)** and expression of key genes involved in benzenoid/phenylpropanoid biosynthesis in *J*. *sambac* cultivar JSDB **(B)**. Abbreviations for enzymes in each catalytic step were shown in red letters. The black numbers in parentheses represent the gene number in *J*. *sambac* genome. The gradient color for each gene represented the gene expression levels at three floral developmental stages (S1, young floral bud stage; S2, mature floral bud stage; S3, initial opening flower stage). These genes were expressed in at least one of the three developmental stages. DAHPS, 3-deoxy-D-arabino-heptulosonate-7-phosphate synthase; DHQS, 3-dehydroquinate synthase; DHD/SDH, dehydroquinate dehydratase/shikimate dehydrogenase; SK, shikimate kinase; EPSPS, 3-phosphoshikimate 1-carboxyvinyltransferase; CS, chorismate synthase; CM, chorismate mutase; PPA-AT, prephenate aminotransferases; ADT/PDT, arogenate dehydratase/prephenate dehydratase; PAL, phenylalanine ammonialyase; BEAT, acetyl-CoA: benzylalcohol acetyltransferase; AAO4, arabidopsis aldehyde oxidase; BAMT, benzoic acid carboxyl methyltransferase; SAMT, salicylic acid methyltransferases.

The results indicated that benzenoids/phenylpropanoids were the predominant components of floral volatile organic compounds (VOCs) (**Table S15**). We identified 16 genes involved in the shikimate pathway, and 13 genes in phenylpyruvate and arogenate pathways (**Figure 3A**; **Table S16**). Our findings indicate that WGD and tandem duplication events played prominent roles in the genes involved in the shikimate pathway, phenylpyruvate and arogenate pathways and benzenoid/phenylpropanoid pathway, which resulted in the high rate of paralog formation. To investigate the biological processes associated with these aroma compounds, we generated RNA-seq data from flowers at the aforementioned developmental stages (**Table S17**) and focused on those genes involved in benzenoid/phenylpropanoid synthesis pathway (**Figure 3B**).

Our analyses revealed benzyl acetate to be one the most prominent compounds among the floral VOCs in single-petal phenotype of *J. sambac*, the emission of which was detected at all three assessed developmental stages (**Figure S8**, **Table S15**). Benzyl acetate is synthesized from benzyl alcohol catalyzed by benzyl alcohol acetyltransferase (BEAT) *via* an acetyl-CoA-dependent reaction. A total of 19 *BEAT* genes were detected in the present *J. sambac* genome (**Figure 3A**), two genes have been generated as a consequence of a WGD event, and 10 are derived from tandem duplication (**Table S16**). Transcriptome analysis revealed that contig913.g1 showed a high expression level at the S1 developmental stage, but contig898.g5 were highly expressed at the S2 stage (**Figure 3B**). Methyl salicylate was also identified as a major component of the floral VOCs in *J. sambac*, and it was found to be present at both S2 and S3 stages (**Figure S8**, **Table S15**). Furthermore, we identified three salicylic acid methyltransferases (*SAMT*s) (**Figure 3A**), with contig995.g4 and contig1566.g7 being highly expressed at the S2 and S3 stages, respectively (**Figure 3B**). Methyl benzoate was similarly identified as a major component of jasmine floral VOCs, which was found to accumulate at stage S3 (**Figure S8**, **Table S15**). In addition, we identified two benzoic acid methyltransferases (*BAMTs*) (**Figure 3A**), and each of them were highly expressed at the S1 stage (**Figure 3B**).

### Genes involved in terpenoid biosynthesis

**Figure 4:**
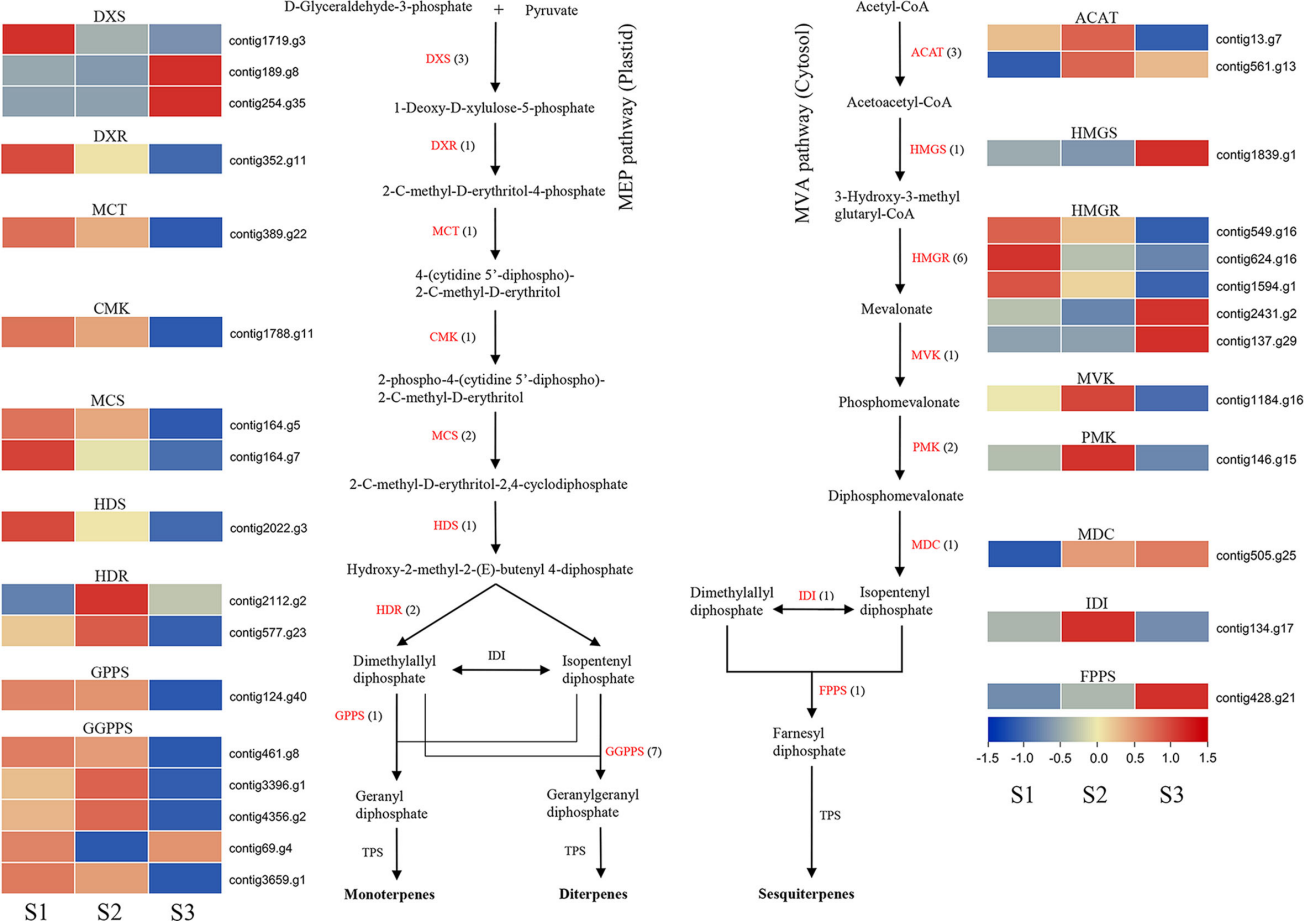
Expression profiles of genes encoding enzymes involved in terpene biosynthesis in *J. sambac* cultivar JSDB. Abbreviations for enzymes in each catalytic step were shown in red letters. The black numbers in parentheses represent the gene number in *J. sambac* genome. The gradient color for each gene represented the gene expression levels at three floral developmental stages (S1, young floral bud stage; S2, mature floral bud stage; S3, initial opening flower stage). DXS, 1-deoxy-D-xylulose 5-phosphate synthase; DXR, 1-deoxy-D-xylulose 5-phosphate reductoisomerase; MCT, 2-C-methyl-D-erythritol 4-phosphate cytidylyltransferase; CMK, 4-(cytidine 5′-diphospho)-2-C-methyl-D-erythritol kinase; MCS, 2-C-methyl-D-erythritol-2,4-cyclodiphosphate synthase; HDS, 4-hydroxy-3-methylbut-2-en-1-yl diphosphate synthase; HDR, 4-hydroxy-3-methylbut-2-en-1-yl diphosphate reductase; GPPS, geranyl diphosphate synthase; GGPPS, geranylgeranyl diphosphate synthase; ACAT, acetyl-CoA acetyltransferase; HMGS, hydroxymethylglutaryl-CoA synthase; HMGR, hydroxymethylglutaryl-CoA reductase; MVK, mevalonate kinase; PMK, phosphomevalonate kinase; MDC, diphospho-MVA decarboxylase; IDI, isopentenyl diphosphate isomerase; FPPS, farnesyl diphosphate synthase.

We found terpenoids as the second major class of compounds among the VOCs produced by *J. sambac* JSDB (**Table S15**), so genes involved in MEP and MVA pathways were identified (**Figure 4**). The results showed that genes involved in both pathways, including *HDR* and *HMGR*, were generated by a WGD event (**Table S18**). Transcriptome analysis revealed that *DXS* (contig1719.g3), *DXR*, *MCT*, *CMK*, *MCS*, *HDS*, *GGPPS* (contig461.g8, contig69.g4 and contig3659.g1) and *HMGR* (contig549.g16, contig624.g16 and contig1594.g1) were highly expressed at the S1 stage (**Figure 4**), whereas *HDR*, *GGPPS* (contig3396.g1 and contig4356.g2), *ACAT*, *MVK*, *PMK* and *IDI* showed high expression at the S2 stage (**Figure 4**). At the same time, *DXS* (contig189.g8 and contig254.g35), *HMGS*, *HMGR* (contig2431.g2 and contig137.g29), *MDC* and *FPPS* tended to be more prominently expressed during the S3 stage (**Figure 4**).

**Figure 5:**
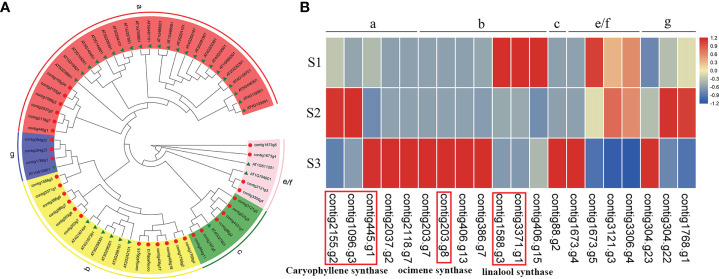
The phylogenetic tree and expression of *TPS* genes in *J*. *sambac* cultivar JSDB. **(A)** The maximum likelihood phylogenetic tree of TPS protein identified in *J*. *sambac*. *J*. *sambac* (red circle) and *A*. *thaliana* (green triangle) were shown in the tree with corresponding gene ID, respectively. **(B)** The expression of *TPSs* at three different floral developmental stages (S1, young floral bud stage; S2, mature floral bud stage; S3, initial opening flower stage). Note the genes were expressed in at least one stage.

In the MVA and MEP pathways, terpene synthases (TPSs) are responsible for the final catalytic reaction in the generation of terpenoid compounds. On the basis of the assembled genomes of *J. sambac* JSDB, we identified 31 TPSs. Phylogenetic analysis revealed that these TPS genes were clustered into five discrete groups, namely TPS-a (6), TPS-b (12), TPS-c (6), TPS-e/f (4) and TPS-g (3) (**Figure 5A**). Linalool was identified as the major component of monoterpenes in the aroma compounds of *J. sambac* JSDB, and was found to be accumulated at the S3 stage (**Figure S8** and **Table S15**). We identified two linalool synthase genes in the present genome, and each of them was highly expressed at the S1 stage (**Figure 5B**). β-Ocimene was another major monoterpene in *J. sambac* JSDB and presented in the S3 stage, and it was synthesized by contig203.g8 (**Figure 5B** and **Table S15**). The contig203.g8 showed a high expression level at the S3 stage, and its expression pattern was consistent with β-Ocimene emission (**Figure 5B**; **Table S15**). Caryophyllene was found to be the major sesquiterpene in *J. sambac* JSDB, and the emission of which was detected at stages S2 and S3 (**Figure S8** and **Table S15**). Furthermore, we identified three caryophyllene synthase genes, among which, contig2155.g2 and contig1096.g3 were characterized by high expression levels at the S2 stage, while contig445.g1 was highly expressed at the S3 stage (**Figure 5B**).

In addition to *TPS* genes, we identified a group TFs involved in the biosynthesis of terpenes (Shang et al., 2020). Screening of the 3000-bp regions upstream of all *TPS* genes in *J. sambac* revealed that defense and stress responsive elements, including MYB-, bHLH-, and WRKY-binding elements, were significantly enriched (**Table S19**). In the newly assembled *J. sambac* genome, we identified a total of 122 MYB, 122 bHLH, and 69 WRKY genes (**Figures S9A**, **S10**, **S12A**). R2R3-MYB subgroup 4, 6, and 7 genes have been shown to regulate phenylpropanoid metabolism in different species (Dubos et al., 2010). In the present *J. sambac* genome, we identified eight genes from the subgroups 4, 6, and 7 (**Figure S9A**), and transcriptional analysis revealed that among these genes, the subgroup 4 genes were highly expressed at the S3 stage, whereas the expression of subgroup 6 and 7 genes was more pronounced at stage S1 (**Figure S9B**). Similarly, most of the bHLH and WRKY TFs were highly expressed at the S1 stage (**Figures S11**, **S12B**).

## Discussion

*J. samba*c is one of the most popularly cultivated ornamental plant species in many countries. It has been cultivated for over 2,000 years in China because of its attractive flower scent, its usage in traditional Chinese medicine, and high value in the famous ‘jasmine tea’. In the study on *J. sambac* cultivar ‘Trifoliatum’ genome assembly, a set of Nanopore long reads (49.00 Gb, ~96×), Illumina paired-end short reads (55.97 Gb, ~110×) and Hi-C data (46.5 Gb, ~91×) were generated (Xu et al., 2021). In another study, they generated 15.35 Gb of HiFi PacBio (single-petal jasmine) and 12.11 Gb of PacBio Sequel (double-petal jasmine) with an estimated coverage depth of over 30-folds for both genomes (Wang et al., 2022). Here, a draft genome of *J. samba*c cultivar JSDB comprising 520.80 Mb was assembled based on Pacbio and Illumination sequencing technologies. Furthermore, combining genomic and transcriptomic analyses, we gained deep insight into heat stress tolerance and aroma compound biosynthesis in the single-petal flowers of *J. samba*c.

TEs play a significant role in genome expansion and evolution, which could lead to an increase in genome size (Bennetzen and Wang, 2014). Based on transposition mechanism, TEs can be broadly divided into two major classes, namely, class I (retrotransposons) and class II (DNA transposons) (Levin and Moran, 2011). Of these, retrotransposons, especially the LTR-RT class, are the most abundant in plant genomes (Wicker et al., 2007). In this study, numerous repetitive elements in the genome (49.01%) were detected, among which, LTR retrotransposons (accounting for 20.56% of the repetitive elements) were the most abundant (**Table 2**). High content of repetitive elements is a common characteristic among the genomes of plant species. For example, in the *Lonicera japonica* genome, transposable elements occupy 58.2% of the genome, of which 45.6% being LTRs (Pu et al., 2020). Similarly, 53.27% of the *Isatis indigotica* genome is repetitive sequences, with LTRs constituting 30.09% (Kang et al., 2020). However, the repetitive elements in the genome of *J. sambac* cultivar JSDB was higher than that of the *J. sambac* cultivar ‘Trifoliatum’ (Xu et al., 2021). In this study, we detected a recent burst of LTR-RTs in the genome (**Figure S5**), a phenomenon that has also been identified in the genomes of *Lonicera japonica*, *Chrysanthemum nankingense* and *Chimonanthus praecox* (Song et al., 2018; Pu et al., 2020; Shang et al., 2020). Consequently, these findings indicate that LTR-RTs may make an important contribution to an increase in the genome size of *J. sambac*.

Previous chloroplast genome and genome analyses have placed *J. sambac* in the Oleaceae family (Qi et al., 2020; Xu et al., 2021). Here, a phylogenomic tree based on a comparison of the *J. sambac* genome with that of 14 other plant species was constructed (**Figure 1C**). We accordingly established that *J. sambac* is closely related to *O. fragrans* and *O. europaea*, belonging to the Oleaceae family (Unver et al., 2017; Yang et al., 2018). WGD plays a central role in plant genome evolution, as it generally leads to a sudden increase in genome size (Panchy et al., 2016; Wendel et al., 2016). Previous research showed that there are two WGDs in *O. europaea* (Unver et al., 2017). In this study, we revealed that the *J. sambac* JSDB genome has only undergone an ancient WGD event, and there was a recent WGD event in the *O. europaea* genome that distinguishes *O. europaea* from *J. sambac* (**Figure 1B**). Therefore, this is a plausible explanation for the size of *O. europaea* genome is larger than *J. sambac* genome (1.48 Gb *vs* 520.8 Mb). Thus, the present sequencing of the JSDB genome provides significant insights for further genomic studies on *J. sambac*, including phenotypic diversity and evolution.

Heat stress generally damages photosynthetic activity and reduces cell division and compromises plant growth (Hasanuzzaman et al., 2013). So it is important to elucidate the molecular mechanism involved in the heat stress response. Here, we identified 92 TFs and 206 genes related to heat stress tolerance in *J. sambac* JSDB, and we also found that some gene families expanded in *J. sambac* (**Tables S12**, **S13**). These will provide useful information for studying the complex transcriptional regulatory networks involved in heat stress response in *J. sambac*. As well known, heat shock transcription factors (Hsf) played a critical role in heat stress response, and *HsfA1s* served as ‘master regulators’ during the process (Ohama et al., 2017). In *Arabidipsis* and tomato, the mutation of *HsfA1s* genes resulted in the reduced induction of heat stress responsive genes and heat stress sensitive phenotypes (Mishra et al., 2002; Yoshida et al., 2011; Fragkostefanakis et al., 2016). *DREB2A* is a plant-specific TF involved in heat stress response, which is directly regulated by *HsfA1s* genes (Ohama et al., 2016). In the present study, both *HsfA1s* and *DREB2A* had highly expressed levels in the S1 stage, and both of them displayed a similar expression profile (**Figures 2**, **S7**). These indicated that *HsfA1s* and *DREB2A* perhaps play a vital role in jasmine’s heat stress response.

Basing on the assembled genome, and using the terpenoid biosynthesis genes in *A. thaliana* and phenylpropanoid biosynthesis genes in *Petunia hybrida* as baits, the homologs genes in *J. sambac* were identified, which revealed a notable duplication of particular genes in *J. sambac*, particularly *PAL*, *BEAT* and *TPS* genes (**Tables S16**, **S19**). In the *Chimonanthus praecox* genome, it has been established that the expansion of *BEAT* and *TPS* genes were attributed to tandem duplication (Shang et al., 2020). Therefore, it can be reasonably speculated that a tandem duplication event has influenced the evolution of *PAL*, *BEAT* and *TPS* genes in *J. sambac* JSDB.

To analyze the genes that contribute to aroma compound biosynthesis in *J. sambac*, we performed comparative transcriptome analysis in combination with metabolome studies. PAL enzymes play a vital role in the initial step of the phenylpropanoid pathway by deaminating l-Phe to yield *trans*-cinnamic acid (Achnine et al., 2004). In this study, there were four *PAL* genes highly expressed at the S3 stage (**Figure 3B**), a pattern of expression that is similar to a former report (Bera et al., 2017). We detected that methyl benzoate accumulated at the S3 stage, whereas the two identified *BAMT* genes were highly expressed at the S1 stage (**Figure 3B**, **Table S15**), indicating that these genes may be actively expressed at the S1 stage in preparation for the following release of methyl benzoate during the S3 stage. The production and diversification of terpenes is mainly determined by the *TPS* family genes and their transcription levels (Chen et al., 2011; Vranová et al., 2013). In this study, we revealed a dynamic expression of *TPSs* (**Figure 5C**), which may account for the observed diversification of the terpene profiles. Within the *TPS* gene family, the members of the *TPS*-b subfamily are involved in the synthesis of monoterpenes (Tholl, 2006; Chen et al., 2011). In the present study, we also established that two linalool synthase genes were highly expressed at the stage S1, whereas linalool was observed to be accumulated at stage S3 (**Figures 5**, **S8**, **Table S15**). This is consistent with the findings of previous studies that have revealed a marked increase in linalool levels coinciding with the initial opening of jasmine flowers (Yu et al., 2017), thereby indicating that these genes are highly expressed at the S1 stage preparing for the release of linalool during stage S3. However, the monoterpene β-Ocimene was only detected in the S3 stage. This is consistent with the expression pattern of the gene (*TPS*-b subfamily) being responsible for β-Ocimene biosynthesis (**Figure 5B**, **Table S15**).

TFs, including those in the MYB (Reddy et al., 2017), bHLH (Hong et al., 2012) and WRKY (Spyropoulou et al., 2014) families, are involved in the regulation of terpenoid biosynthesis (Shang et al., 2020). In the present study, we detected multiple MYB-, bHLH- and WRKY-binding elements in the promoter sequences of *JsTPS* genes (**Table S19**). Our results indicate that these TFs may play a key role in regulating the expression of *TPS* genes and will provide valuable information for further studies, in which intend to examine the co-expression patterns of TFs and aroma compound pathway genes.

## Conclusion

In summary, in the present study, we generated a draft genome of a single-petal phenotype of *J. sambac*, a culturally and commercially important plant in the Oleaceae family. The newly assembled genome will provide a solid foundation for further research of resistance to abiotic stresses, aroma compound biosynthesis and genomic evolution. Moreover, the genome will contribute to gain a more comprehensive understanding of the molecular mechanisms underlying heat stress tolerance, flower development and its scent formation.

## Data availability statement

The original contributions presented in the study are publicly available. This data can be found here: NCBI, BioProject PRJNA690159 and BioSample SAMN17245799. The genome assembly and annotation files are available publicly at FigShare (https://doi.org/10.6084/m9.figshare.17030054.v1).

## Author contributions

YD and XQ managed and organized the project. HW and HC collected plant samples and performed experiments. XQ and SC contributed to genome assembly, genome annotation and evolutionary analyses. XQ, HW, JF and ZQ analyzed the data. XQ, IB and YD wrote and revised the manuscript. All authors contributed to the article and approved the submitted version.

## Funding

This work was financially supported by the National Natural Science Foundation of China (Grant No.: 31772338), and the Basic Scientific Research Business Special Project of Jiangsu Academy of Agricultural Sciences (0090756100ZX).

## Acknowledgments

We thank the staffs of the Central Laboratory at Jiangsu Academy of Agricultural Sciences for their help in HS-SPME and GC-MS analysis.

## Conflict of interest

The authors declare that the research was conducted in the absence of any commercial or financial relationships that could be construed as a potential conflict of interest.

## Publisher’s note

All claims expressed in this article are solely those of the authors and do not necessarily represent those of their affiliated organizations, or those of the publisher, the editors and the reviewers. Any product that may be evaluated in this article, or claim that may be made by its manufacturer, is not guaranteed or endorsed by the publisher.
