# Supplementary material for: The genome of single-petal jasmine (Jasminum sambac) provides insights into heat stress tolerance and aroma compound biosynthesis
Source: Front Plant Sci. 2022 Oct 19;13:1045194. doi: 10.3389/fpls.2022.1045194 (PMC9627619; doi:10.3389/fpls.2022.1045194)
Supplement: Supplementary file 1 [file DataSheet_1.doc]

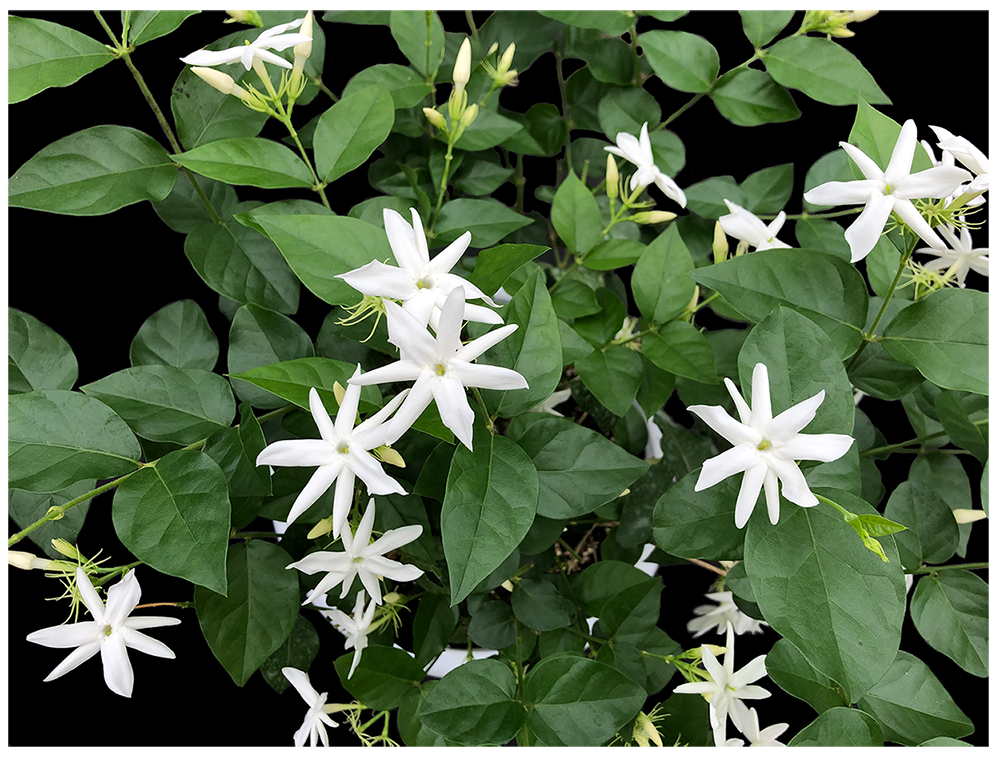


**Figure S1. The plant of *J. sambac* cultivar ‘Danbanmoli’ (JSDB).**


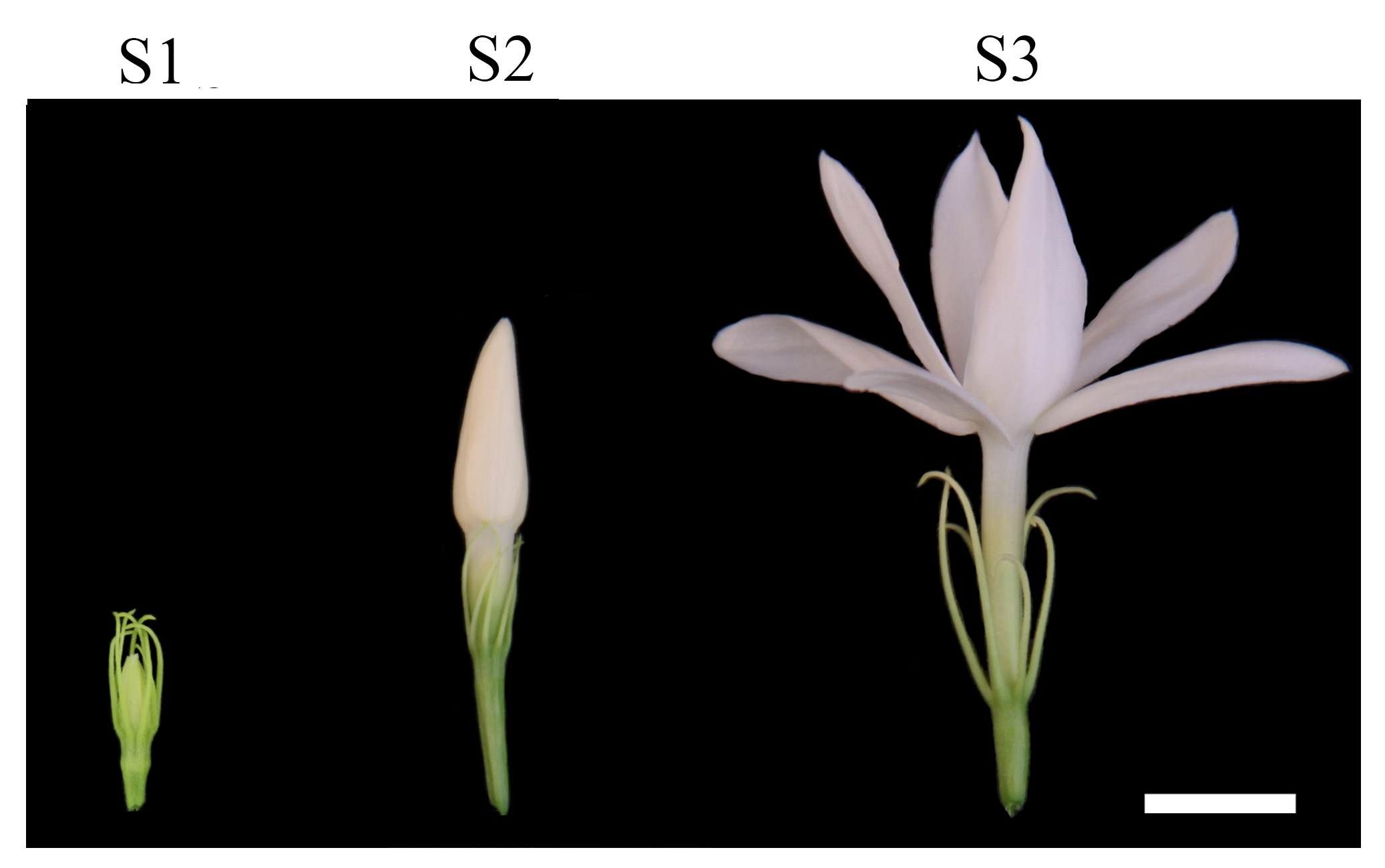


**Figure S2. The morphology of sampled floral buds at different developmental stages.** S1, young floral bud stage; S2, mature floral bud stage; S3, initial opening flower stage. Bar= 10 mm.


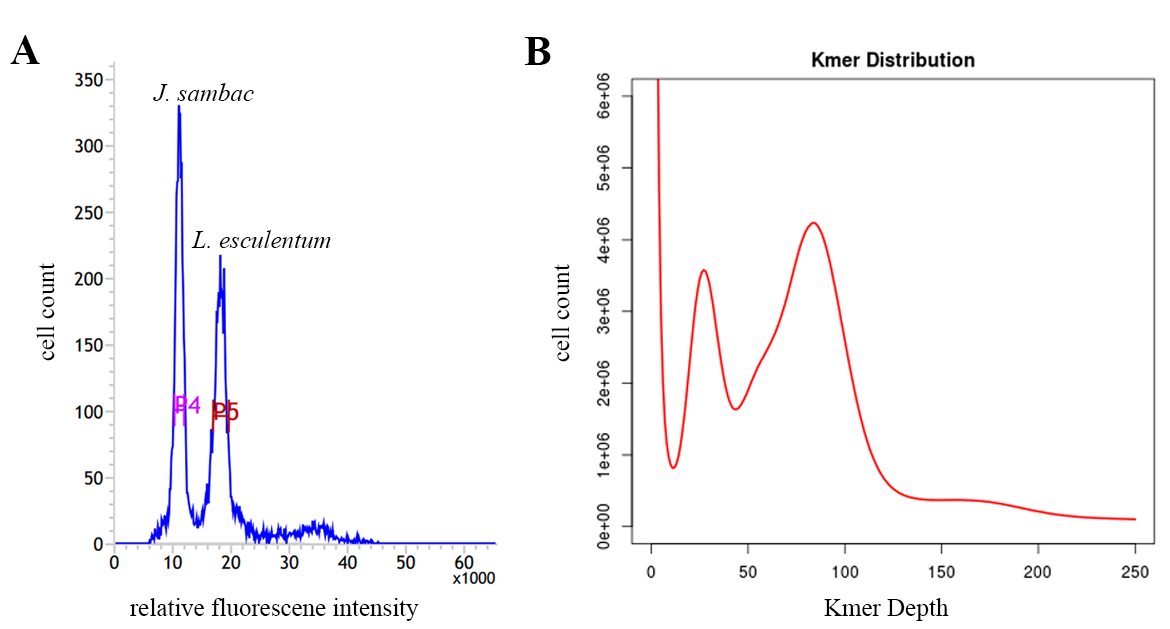


**Figure S3. Estimation of *J. sambac* cultivar JSDB genome size.** **(A)** Estimation of *J. sambac* genome size based on flow cytometer analysis. **(B)** The distribution of *J. sambac* 17-mers. Two peaks are observed (at 42× and 84×, respectively) indicating the heterozygosity in *J. sambac*.

Illumina reads

(98× of the estimated genome size)

DBG2LOC

Contigs

(genome size=521 Mb, N50=145 kb)

Scaffold

(genome size=521 Mb, N50=145 kb)

Pacbio reads

(28× of the estimated genome size)

Platanus (Contig)

(total length=814 Mb, N50=477 bp)

**Figure S4. Integrated work-flow for the assembly of the *J. sambac* cultivar JSDB genome.**


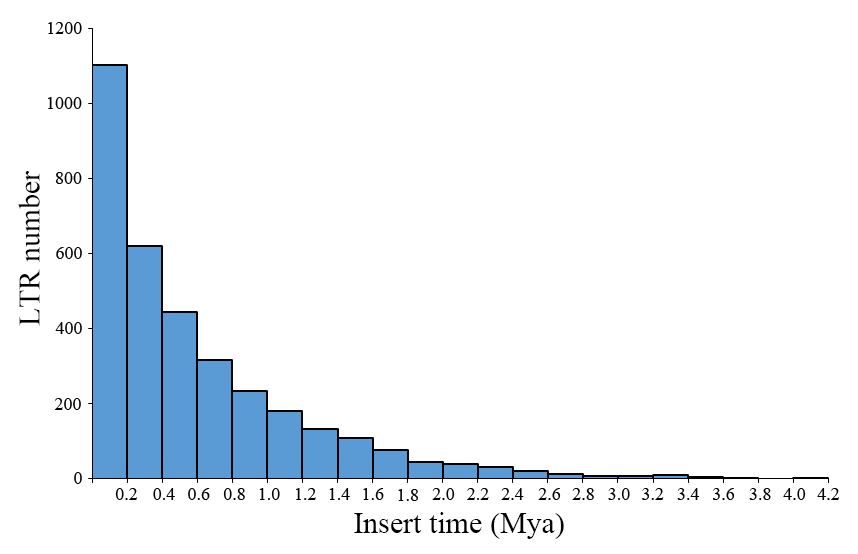


**Figure S5. The insert time of LTR retrotransposons in *J. sambac* cultivar JSDB.**


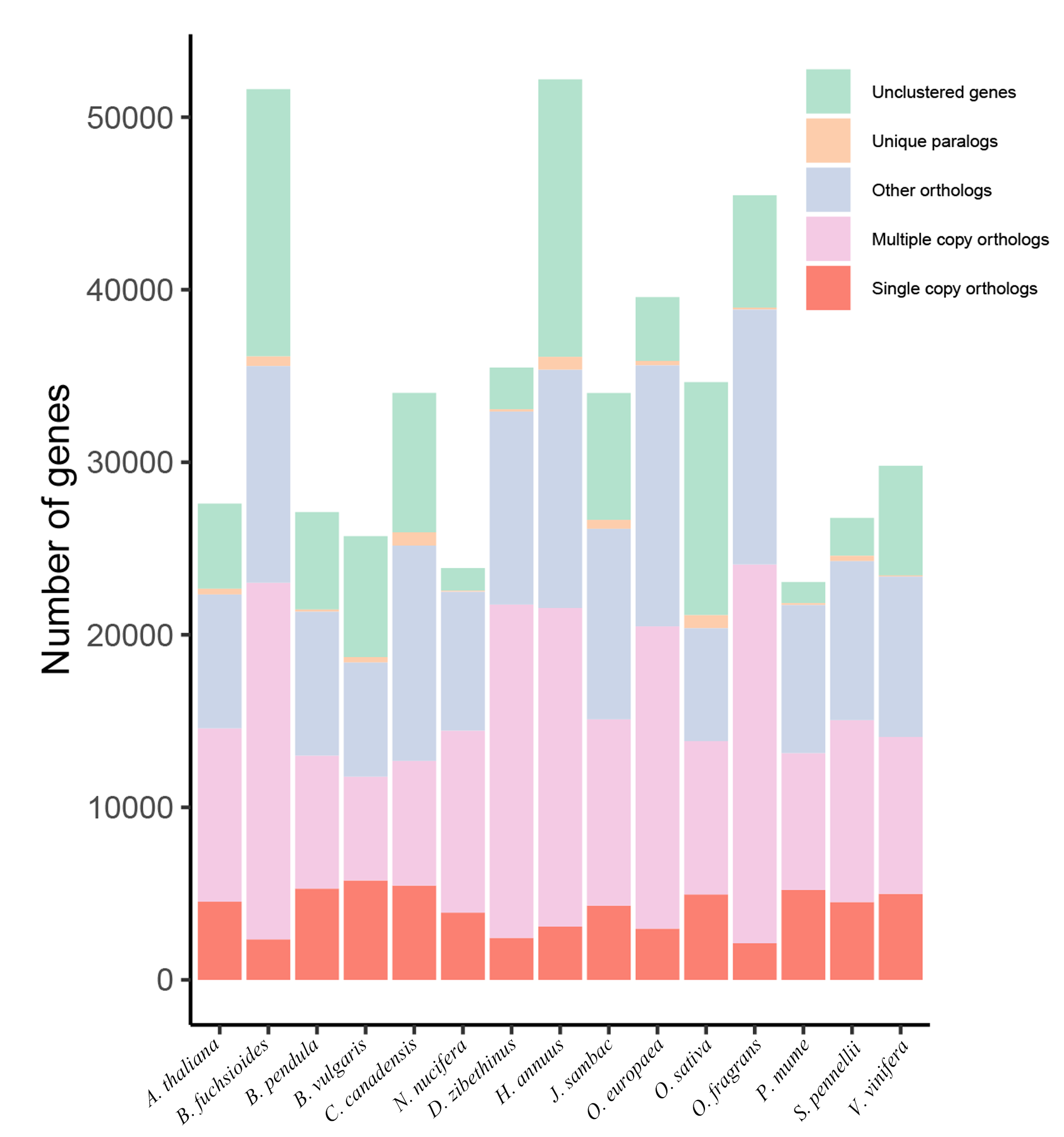


**Figure S6. Distribution of genes and gene families among the plant species defined by OrthoFinder.**


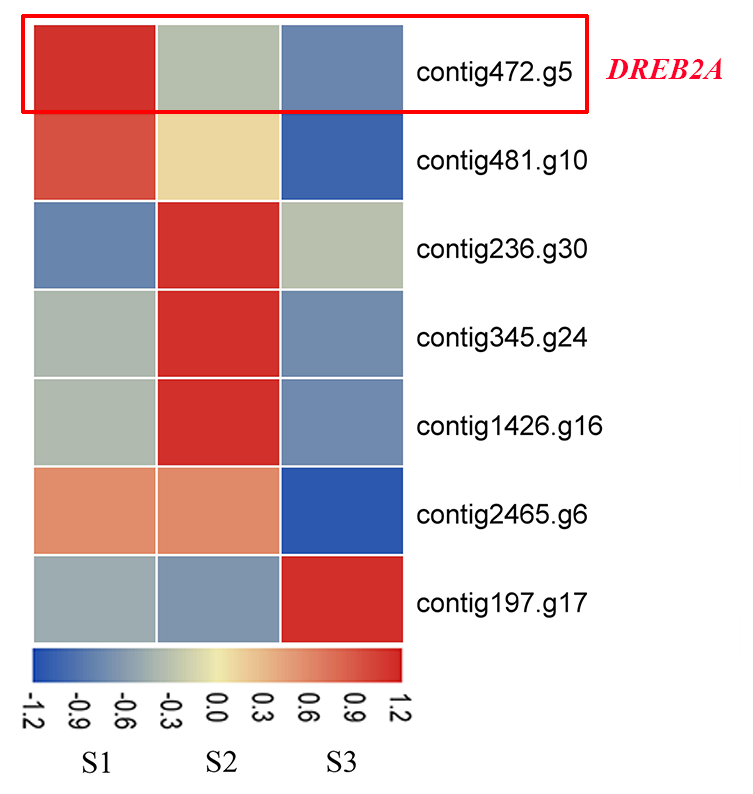


**Figure S7. The expression of *DREB2s* in the three floral developmental stages of *J. sambac* cultivar JSDB.** S1, young floral bud stage; S2, mature floral bud stage; S3, initial opening flower stage.


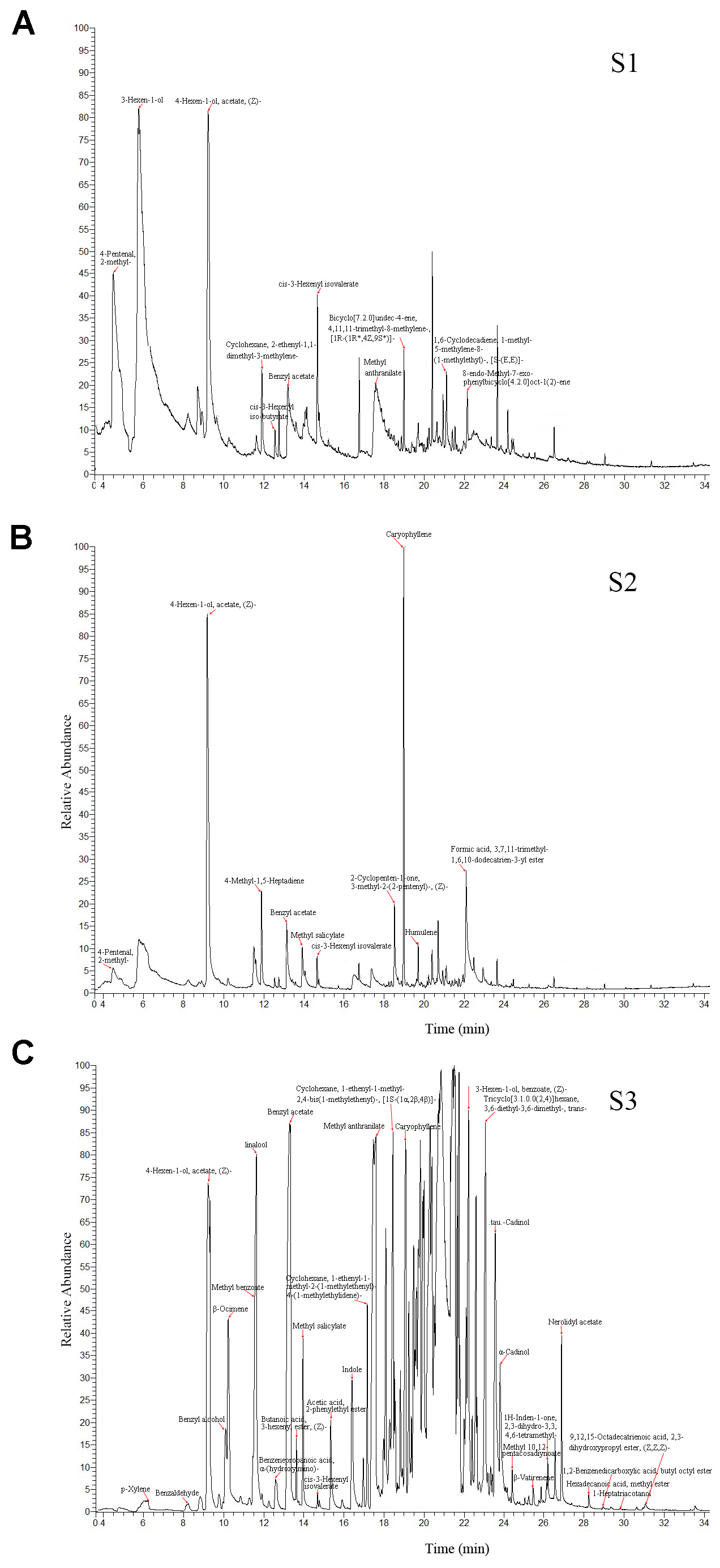


**Figure S8. Gas chromatogram of aroma compounds from the flowers of *J. sambac* cultivar JSDB.** **(A)** S1. **(B)** S2. **(C)** S3. S1, young floral bud stage; S2, mature floral bud stage; S3, initial opening flower stage.


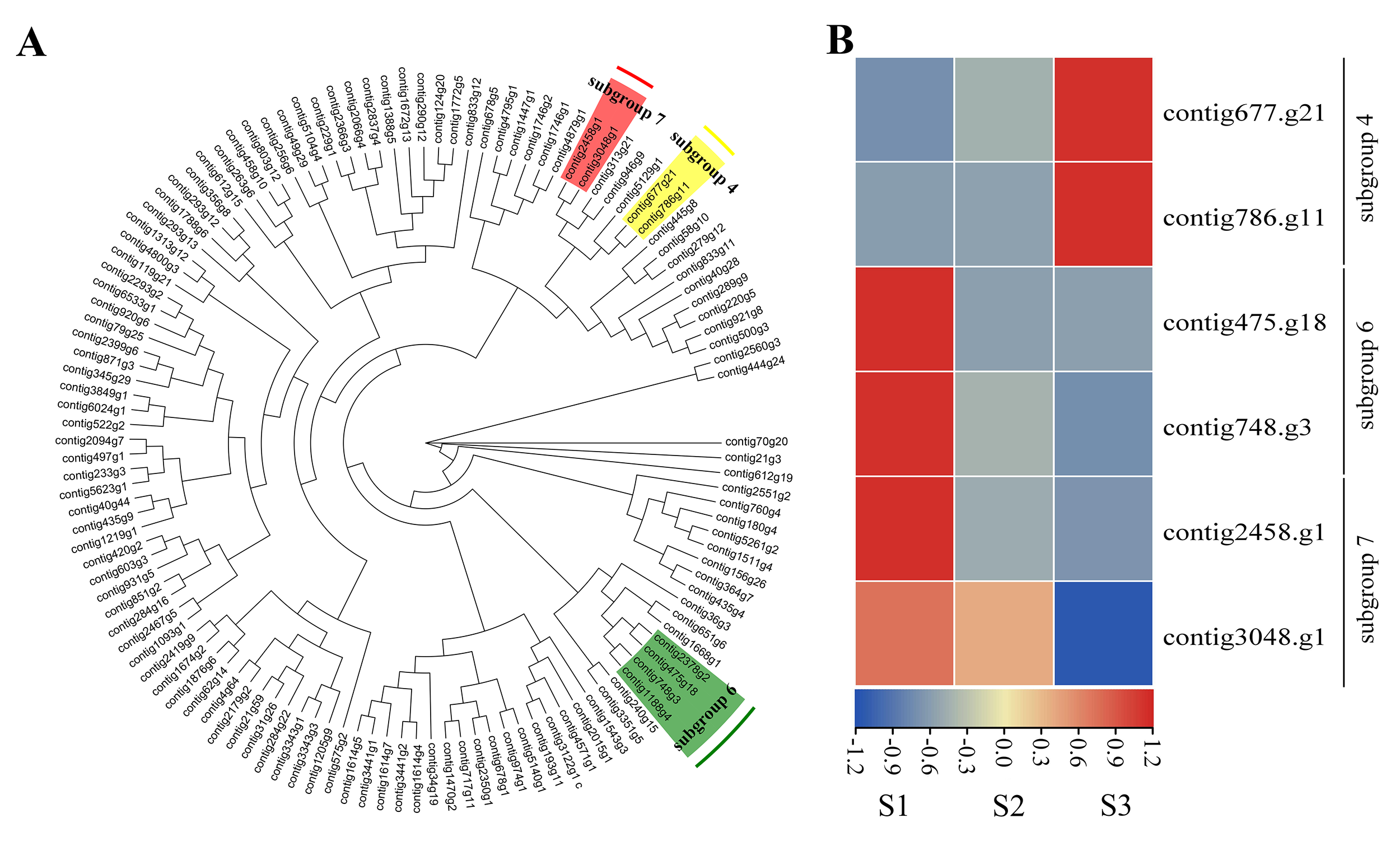


**Figure S9. Phylogenetic relationship of MYB transcriptional factors identified in the *J. sambac* cultivar JSDB genome (A) and the expression of six genes in the subgroup 4, 6 and 7 (B).** S1, young floral bud stage; S2, mature floral bud stage; S3, initial opening flower stage.


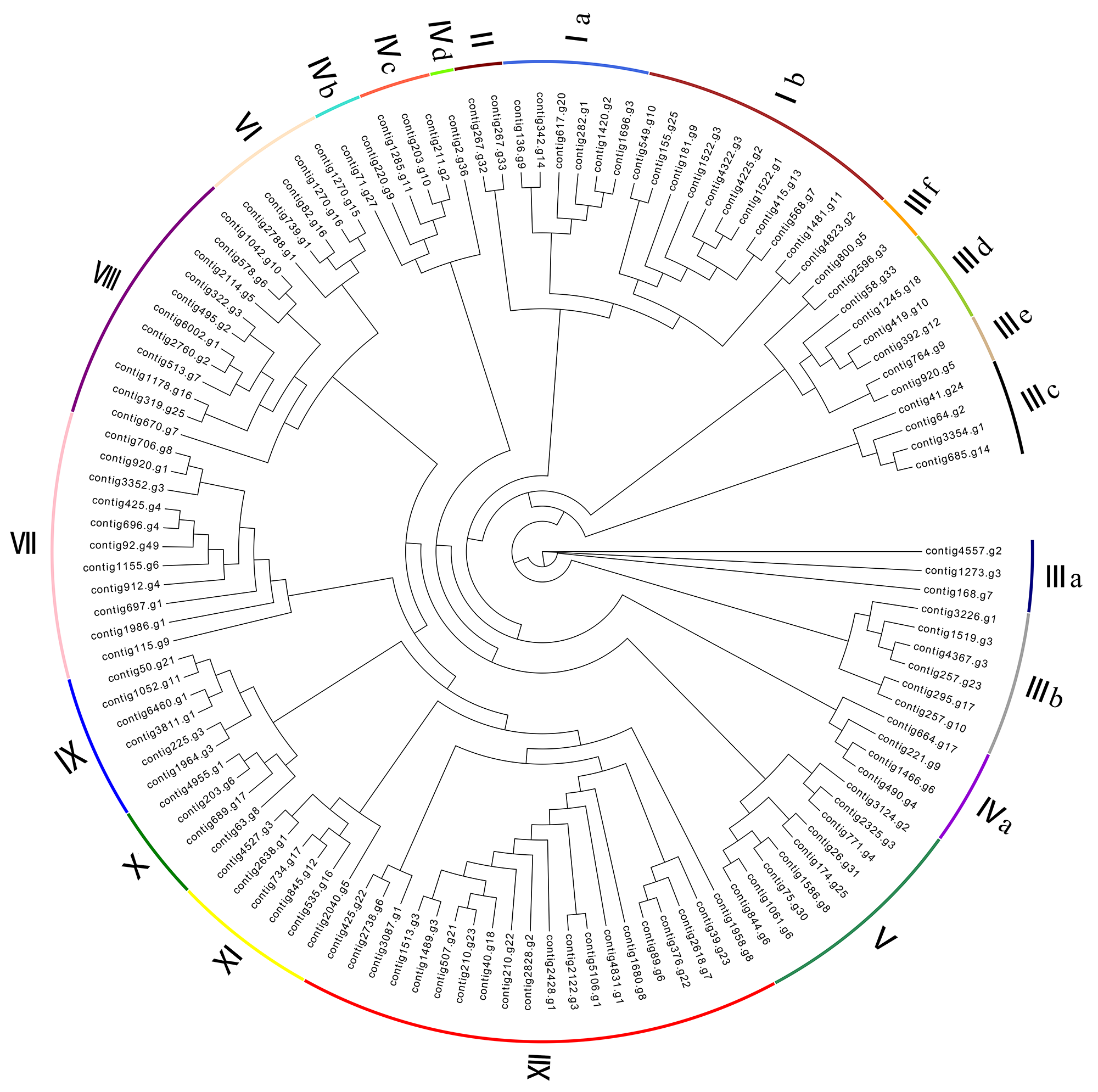


**Figure S10. Phylogenetic relationship of bHLH transcriptional factors identified in the *J. sambac* cultivar JSDB genome.**


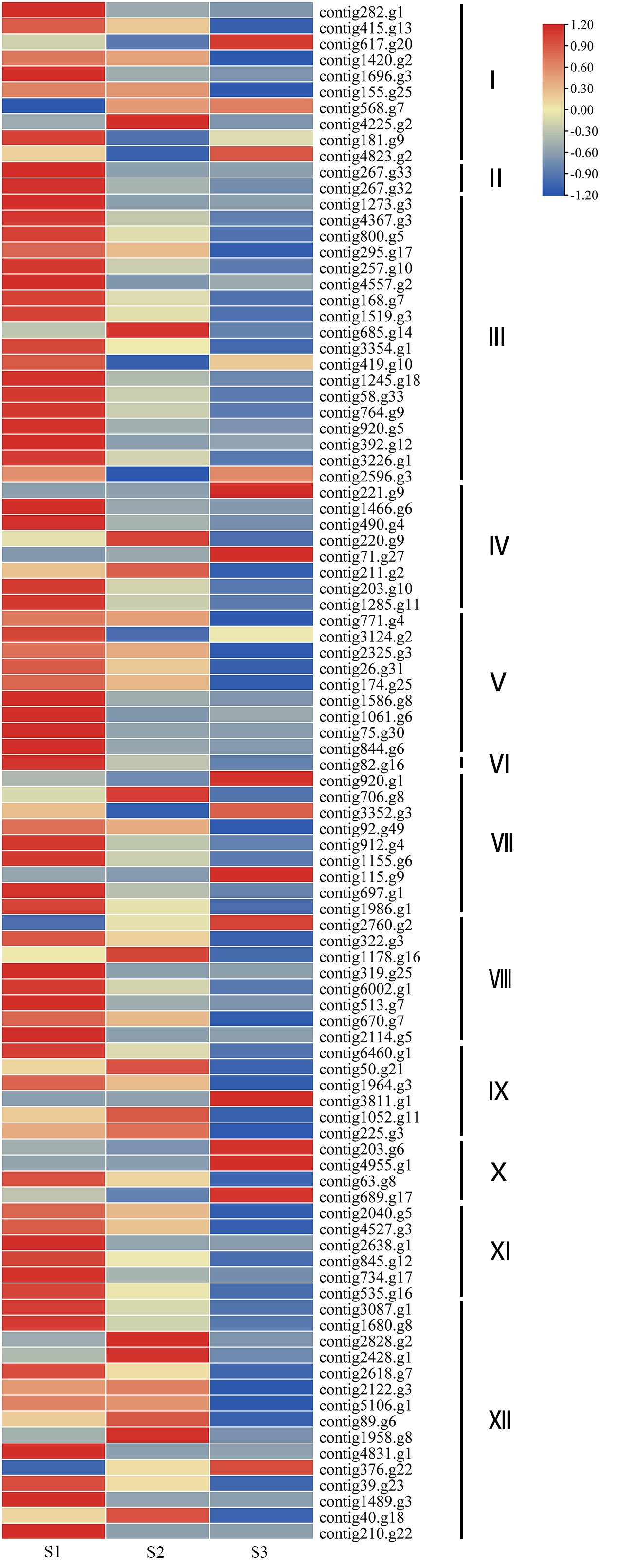


**Figure S11. The expression of bHLH genes in the flowers at different development stages of *J. sambac* cultivar JSDB.** S1, young floral bud stage; S2, mature floral bud stage; S3, initial opening flower stage.


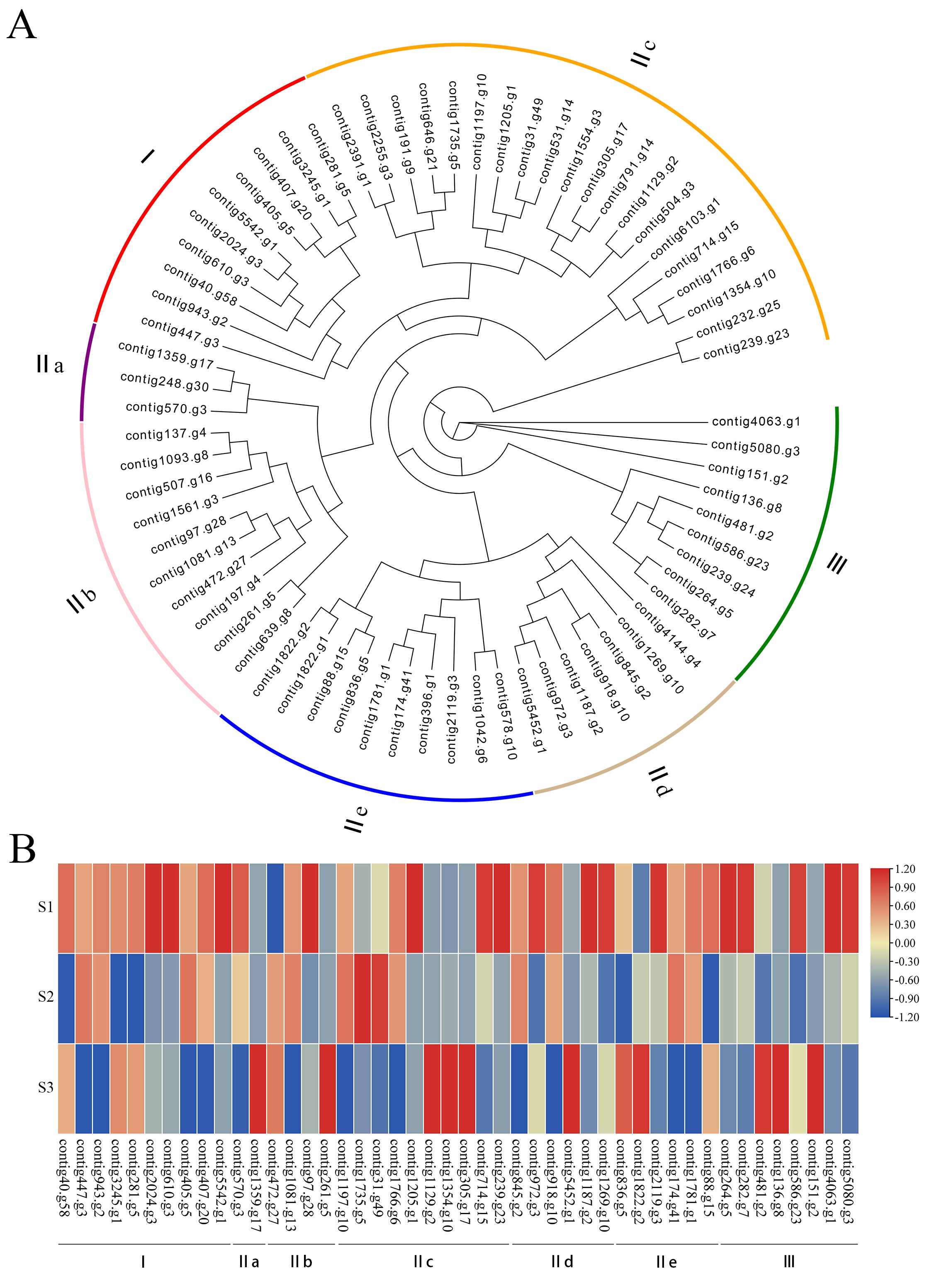


**Figure S12. Phylogenetic relationship of WRKY transcriptional factors identified in the genome of *J. sambac* cultivar JSDB (A) and the expression of WRKY genes in the different developmental stages (B).** S1, young floral bud stage; S2, mature floral bud stage; S3, initial opening flower stage.
