# Supplementary material for: The genome of single-petal jasmine (Jasminum sambac) provides insights into heat stress tolerance and aroma compound biosynthesis
Source: Front Plant Sci. 2022 Oct 19;13:1045194. doi: 10.3389/fpls.2022.1045194 (PMC9627619; doi:10.3389/fpls.2022.1045194)
Supplement: Supplementary file 2 [file DataSheet_2.doc]

**Table S1. Overview of sequencing strategies.**

| Sample type | DNA | DNA | RNA |
| --- | --- | --- | --- |
| Library function | Survey/Assembly | Assembly | Annotation |
| Library | PE400 | S20K | PE400 |
| Library insert size | 400 bp | 20,000 bp | 400 bp |
| Sequencing platform | Illumina HiSeq | PacBio Sequel | Illumina HiSeq |
| Sequencing mode | Paired-end, 2×150 bp | Standard | Paired-end, 2×150 bp |

**Table S2. Genomes of representative plant species used in phylogenomics and comparative genomics analyses.**

| Index | Plant taxon | Scientific name | Family |
| --- | --- | --- | --- |
| 1 | Arabidopsis | *Arabidopsis thaliana* | Brassicaceae |
| 2 | Fuchsia begonia | *Begonia fuchsioides* | Begoniaceae |
| 3 | Common beet | *Beta vulgaris* | Chenopodiaceae |
| 4 | Silver birch | *Betula pendula* | Betulaceae |
| 5 | Eastern redbud | *Cercis canadensis* | Leguminosae |
| 6 | Durian | *Durio zibethinus* | Bombacaceae |
| 7 | Sunflower | *Helianthus annuus* | Asteraceae |
| 8 | Water lily | *Nelumbo nucifera* | Nymphaeaceae |
| 9 | Rice | *Oryza sativa* | Poaceae |
| 10 | Mei | *Prunus mume* | Rosaceae |
| 11 | Wide tomato | *Solanum pennellii* | Solanaceae |
| 12 | Grape | *Vitis vinifera* | Vitaceae |
| 13 | Wide olive | *Olea europaea* | Oleaceae |
| 14 | Sweet osmanthus | *Osmanthus fragrans* | Oleaceae |
| 15 | Jasmine | *Jasminum sambac* | Oleaceae |

**Table S3. Results of genome size of *J. sambac* cultivar JSDB based on flow cytometry.**

| Sample No. | Peak of  *J. sambac* | Peak of  *L. esculentum* | Ratio | 1 C-value  (pg) | 1 C size  (Mbp) |
| --- | --- | --- | --- | --- | --- |
| 1 | 10,962 | 18,094 | 0.6058 | 0.5934 | 580.3911 |
| 2 | 9,846 | 16,240 | 0.6063 | 0.5939 | 580.8170 |
| 3 | 10,085 | 16,396 | 0.6151 | 0.6025 | 589.2553 |
| Average |  |  |  | 0.5966±0.0029 | 583.4878±2.8864 |

**Table S4. The information of reads sequenced by Illumina HiSeq.**

| Sample type | DNA | RNA |  |
| --- | --- | --- | --- |
| Library function | Survey | Annotation |  |
| High quality reads | 353,723,590 | 91,704,398 |  |
| Raw reads | 362,044,902 | 92,760,952 |  |
| High quality reads % | 97.70% | 98.86% |  |
| High quality data (bp) | 52,362,850,208 | 13,813,054,258 |  |
| Raw data (bp) | 54,668,780,202 | 14,006,903,752 |  |
| High quality data % | 95.78% | 98.62% |  |

**Table S5. Results of 17-mer of *J. sambac* cultivar JSDB.**

|  | Property |  | *J. sambac* |
| --- | --- | --- | --- |
|  | K-mer |  | 17 |
|  | K-mer num |  | 398,562,836 |
|  | K-mer peak depth | (×) | 84 |
|  | Low frequency K-mer num (≦2) | | 45,076,550 |
|  | Heterozygosity(%) |  | 0.844 |
|  | Repetitive 17-mer fraction | | 10.49% |
|  | Avg. Read Length | (bp) | 148.03 |
|  | Total Read Num. |  | 353,723,590 |
|  | Genome size (bp) |  | 555,453,693 |

**Table S6. The information of reads sequenced by PacBio Sequel.**

| Property | *J. sambac* |
| --- | --- |
| Total sequence number | 1,609,052 |
| Total sequence length (bp) | 15,922,933,902 |
| GC content (%) | 36.94% |
| N number | 0 |
| N rate | 0 |
| Sequences greater than 1kb | 1,503,120 |
| Shortest (bp) | 200 |
| Longest (bp) | 71,577 |
| N20 (bp) | 21,181 |
| N50 (bp) | 14,074 |
| N90 (bp) | 5,931 |

**Table S7. Assessing the completeness of the assembled genome of *J. sambac* cultivar JSDB with BUSCO.**

| BUSCO notation | Number | Percent |
| --- | --- | --- |
| Complete BUSCOs (C) | 1,306 | 95.0% |
| Complete and single-copy BUSCOs (S) | 1,193 | 86.8% |
| Complete and duplicated BUSCOs (D) | 113 | 8.2% |
| Fragmented BUSCOs (F) | 21 | 1.5% |
| Missing BUSCOs (M) | 48 | 3.5% |
| Total BUSCO groups searched | 1,375 | 100% |

**Table S8. Non-coding RNA genes annotated in the *J. sambac* cultivar JSDB genome.**

| Type | Copy number | Average length (bp) | Total length (bp) | Percentage of genome |
| --- | --- | --- | --- | --- |
| miRNA | 261 | 129 | 33,704 | 6.47E-05 |
| tRNA | 630 | 75 | 47,076 | 9.04E-05 |
| rRNA | 122 | 621 | 75,725 | 0.0001454 |
| snRNA | 781 | 114 | 88,990 | 0.000171 |

**Table S9. Statistics of the functional genes of *J. sambac* cultivar JSDB.**

| Property | *J. sambac* |
| --- | --- |
| Total genes length | 117,517,874 |
| Genes percentage of genome | 22.56% |
| Total genes number | 35,363 |
| Average gene length | 3323.1 |
| Total exons number | 171,817 |
| Average exons per gene | 4.8 |
| Total exons length | 44,987,385 |
| Exons percentage of genome | 8.64% |
| Total exons number with CDS | 166,261 |
| Average exons length with CDS | 218 |
| Average exons length | 261.8 |
| Average introns length | 525.4 |
| Total CDSs length | 36,259,583 |
| CDSs percentage of genome | 6.96% |
| Average CDS length | 1025.3 |

**Table S10. The annotated genes of *J. sambac* cultivar JSDB which can be functionally classified in each corresponding database.**

| Database | Number | Percentage |
| --- | --- | --- |
| NR | 29,802 | 84.27% |
| GO | 13,829 | 39.11% |
| eggNOG | 28,763 | 81.34% |
| KEGG | 7,185 | 20.32% |
| Swiss-Prot | 23,186 | 65.57% |
| Total | 29,921 | 84.61% |

**Table S11. Comparisons of genes and gene families among the investigated plant species**.

| Species | Total genes | Clustered  genes | Species specific genes | Total gene families | Species specific gene family |
| --- | --- | --- | --- | --- | --- |
| *A. thaliana* | 27,602 | 22,670 | 337 | 12,416 | 53 |
| *B. fuchsioides* | 51,620 | 36,132 | 561 | 12,930 | 154 |
| *B. vulgaris* | 25,717 | 18,709 | 320 | 12,484 | 56 |
| *B. pendula* | 27,111 | 21,463 | 127 | 13,274 | 28 |
| *C. canadensis* | 34,018 | 25,940 | 769 | 13,513 | 163 |
| *D. zibethinus* | 35,487 | 33,068 | 132 | 13,223 | 33 |
| *H. annuus* | 52,191 | 36,109 | 750 | 13,583 | 104 |
| *N. nucifera* | 23,865 | 22,554 | 63 | 12,846 | 19 |
| *O. sativa* | 34,643 | 21,135 | 757 | 12,052 | 128 |
| *P. mume* | 23,060 | 21,832 | 110 | 13,086 | 17 |
| *S. pennellii* | 26,775 | 24,579 | 311 | 12,797 | 49 |
| *V. vinifera* | 29,795 | 23,440 | 69 | 13,714 | 15 |
| *O. europaea* | 39,570 | 35,873 | 262 | 14,343 | 43 |
| *O. fragrans* | 45,473 | 38,959 | 114 | 14,733 | 33 |
| *J. sambac* | 35,363 | 26,660 | 519 | 13,542 | 61 |

**Table S12. A summary of heat stress response transcription factor identified in *J. sambac* cultivar JSDB and other species.**

| Genes | *J. sambac* | *A. thaliana* | *H. annuus* |
| --- | --- | --- | --- |
| *Hsf* | 17 | 18 | 32 |
| *DREB2* | 15 | 7 | 21 |
| *NAC* | 34 | 2 | 115 |
| *MBF1C* | 2 | 1 | 4 |
| *GRF7* | 5 | 1 | 8 |
| *bZIP28* | 2 | 1 | 1 |
| *SPLs* | 17 | 11 | 21 |

*Hsf*: heat shock transcription factor; *DREB2*: dehydration-responsive element binding protein 2; *NAC*: NAM, ATAF and CUC; *MBF1C*: multiprotein bridging factor 1C; *GRF7*: growth-regulating factor 7; *bZIP28*: basic leucine zipper 28; *SPL*: squamosa-promoter binding-like.

**Table S13. A summary of heat stress response genes identified in *J. sambac* cultivar JSDB and other species.**

| Genes | *J. sambac* | *A. thaliana* | *H. annuus* |
| --- | --- | --- | --- |
| *HSP* | 73 | 64 | 146 |
| *APX* | 8 | 8 | 9 |
| *CAT* | 6 | 3 | 6 |
| *NF-YA2* | 8 | 1 | 9 |
| *NF-YB3* | 11 | 1 | 21 |
| *DPB3-1/NF-YC10* | 3 | 1 | 1 |
| *CDKA1* | 34 | 1 | 39 |
| *CBK3* | 14 | 1 | 24 |
| *PP7* | 6 | 3 | 2 |
| *RCD1* | 1 | 1 | 0 |
| *DRIP1/2* | 2 | 2 | 3 |
| *ASF1* | 1 | 2 | 2 |
| *CSD1/2* | 3 | 2 | 4 |
| *CCS* | 3 | 1 | 1 |
| *HIT4* | 4 | 1 | 2 |
| *DDM1* | 7 | 1 | 9 |
| *CNGCs* | 21 | 4 | 30 |
| *PLC9* | 1 | 1 | 9 |

*HSP*: heat shock proteins; *APX*: ascorbate peroxidase; *CAT*: catalase; *NF-YA2*: nuclear factor Y subunit A2; *NF-YB3*: nuclear factor Y subunit B3; *DPB3-1/NF-YC10*: DNA polymerase II subunit B3-1/nuclear factor Y subunit C10; *CDKA1*: cyclin-dependent kinase A1; *CBK3*: calmodulin-binding protein kinase 3; *PP7*: protein phosphatase 7; *RCD1*: radical-induced cell death 1; *DRIP1/2*: DREB2A-interacting protein1/2; *ASF1*: anti-silencing function 1; *CSD1/2*: copper/zinc superoxide dismutase 1/2; *CCS*: copper chaperone for SOD1; *HIT4*: heat-intolerant 4; *DDM1*: decrease in DNA methylation 1; *CNGC*: cyclic nucleotide gated channel; *PLC9*: phosphoinositide-specific phospholipase C9.

**Table S14. A summary of Hsf and DREB2 transcription factors identified in the *J. sambac* cultivar JSDB genome.**

| Gene family | Gene ID | Gene description |
| --- | --- | --- |
| Hsf | contig103.g3 | Heat shock transcription factor A1D |
|  | contig2860.g5 | Heat shock transcription factor A1D |
|  | contig1614.g11 | Heat shock transcription factor A1E |
|  | contig379.g23 | Heat shock transcription factor A2 |
|  | contig1474.g6 | Heat shock transcription factor A6B |
|  | contig930.g14 | Heat shock transcription factor A6B |
|  | contig734.g5 | Heat shock transcription factor A7A |
|  | contig3431.g3 | Heat shock transcription factor A7A |
|  | contig3431.g4 | Heat shock transcription factor A7A |
|  | contig997.g1 | Heat shock transcription factor A8 |
|  | contig5.g29 | Heat shock transcription factor B4 |
|  | contig3580.g5 | Heat shock transcription factor B4 |
|  | contig1919.g1 | Heat shock transcription factor B2 |
|  | contig59.g32 | Heat shock transcription factor B3 |
|  | contig4931.g1 | Heat shock transcription factor B-2b |
|  | contig1004.g12 | Heat shock transcription factor C1 |
|  | contig3831.g2 | Heat shock transcription factor C1 |
| DREB2 | contig442.g4 | Dehydration-responsive element-binding protein 2A |
|  | contig472.g5 | Dehydration-responsive element-binding protein 2A |
|  | contig1602.g2 | Dehydration-responsive element-binding protein 2A |
|  | contig4233.g2 | Dehydration-responsive element-binding protein 2A |
|  | contig197.g17 | Dehydration-responsive element-binding protein 2C |
|  | contig236.g30 | Dehydration-responsive element-binding protein 2C |
|  | contig345.g24 | Dehydration-responsive element-binding protein 2C |
|  | contig1426.g16 | Dehydration-responsive element-binding protein 2C |
|  | contig2465.g6 | Dehydration-responsive element-binding protein 2C |
|  | contig5762.g1 | Dehydration-responsive element-binding protein 2C |
|  | contig481.g10 | Dehydration-responsive element-binding protein 2D |
|  | contig50.g11 | Dehydration-responsive element-binding protein 2F |
|  | contig145.g2 | Dehydration-responsive element-binding protein 2F |
|  | contig617.g14 | Dehydration-responsive element-binding protein 2F |
|  | contig6223.g1 | Dehydration-responsive element-binding protein 2F |

**Table S15. Aroma compounds and contents of *J. sambac* cultivar JSDB at different developmental stages.**

| Compound Name | Retention Time (min) | S1 (100%) | S2 (100%) | S3 (100%) |
| --- | --- | --- | --- | --- |
| **Esters** |  |  |  |  |
| 4-Hexen-1-ol, acetate, (Z)- | 9.19 | 29.88±0.01 | 43.99±0.09 | 16.31±0.01 |
| Methyl anthranilate | 17.58 | 18.73±0.01 | — | 21.76±0.03 |
| Benzyl acetate | 13.19 | 4.05±0.01 | 2.84±0.01 | 16.40±0.01 |
| cis-3-Hexenyl isovalerate | 14.66 | 2.55±0 | 2.40±0.01 | 0.08±0 |
| cis-3-Hexenyl iso-butyrate | 12.54 | 0.71±0 | — | — |
| Limonen-6-ol, pivalate | 23.33 | 0.04±0 | — | — |
| Formic acid, 3,7,11-trimethyl-1,6,10-dodecatrien-3-yl ester | 22.13 | — | 4.39±0.01 | — |
| Methyl salicylate | 13.96 | — | 2.85±0.01 | 2.32±0 |
| trans-β-Terpinyl benzoate | 11.61 | — | 1.05±0 | — |
| 3-Hexen-1-ol, benzoate, (Z)- | 22.18 | — | — | 4.35±0.01 |
| Methyl benzoate | 11.46 | — | — | 2.76±0 |
| Acetic acid, 2-phenylethyl ester | 15.34 | — | — | 1.68±0 |
| Nerolidyl acetate | 26.88 | — | — | 1.10±0 |
| Butanoic acid, 3-hexenyl ester, (Z)- | 13.63 | — | — | 0.47±0 |
| 1,2-Benzenedicarboxylic acid, butyl octyl ester | 28.95 | — | — | 0.19±0 |
| Phthalic acid, butyl tetradecyl ester | 27.38 | — | — | 0.16±0 |
| Cyclohexanol, 1-methyl-4-(1-methylethenyl)-, acetate | 9.75 | — | — | 0.14±0 |
| Hexadecanoic acid, methyl ester | 28.24 | — | — | 0.12±0 |
| Methyl 10,12-pentacosadiynoate | 24.42 | — | — | 0.06±0 |
| Hexadecanoic acid, ethyl ester | 29.33 | — | — | 0.04±0 |
| 9,12,15-Octadecatrienoic acid, 2,3-dihydroxypropyl ester, (Z,Z,Z)- | 31.07 | — | — | 0.02±0 |
| **Alcohols** |  |  |  |  |
| 3-Hexen-1-ol | 5.77 | 22.44±0.07 | — | — |
| 1-Dodecanol, 3,7,11-trimethyl- | 24.19 | 0.86±0 | — | — |
| 3,7-Octadiene-2,6-diol, 2,6-dimethyl- | 12.54 | — | 0.74±0 | — |
| linalool | 11.64 | — | — | 4.38±0 |
| .tau.-Cadinol | 23.58 | — | — | 4.03±0 |
| Benzyl alcohol | 10.10 | — | — | 2.20±0.01 |
| α-Cadinol | 23.82 | — | — | 0.57±0 |
| Bicyclo[4.4.0]dec-2-ene-4-ol, 2-methyl-9-(prop-1-en-3-ol-2-yl)- | 25.85 | — | — | 0.02±0 |
| 1-Heptatriacotanol | 29.80 | — | — | 0.01±0 |
| **Terpenes** |  |  |  |  |
| 1,6-Cyclodecadiene, 1-methyl-5-methylene-8-(1-methylethyl)-, [S-(E,E)]- | 21.10 | 1.60±0 | — | — |
| γ-Muurolene | 20.93 | 0.58±0 | — | — |
| Bicyclo[7.2.0]undec-4-ene, 4,11,11-trimethyl-8-methylene-,[1R-(1R*,4Z,9S*)]- | 18.98 | 0.50±0 | — | — |
| 8-endo-Methyl-7-exo-phenylbicyclo[4.2.0]oct-1(2)-ene | 22.13 | 0.45±0 | — | — |
| 4-Methyl-1,5-Heptadiene | 11.89 | — | 25.85±0.15 | — |
| Caryophyllene | 18.99 | — | 6.63±0.02 | 1.58±0 |
| Humulene | 19.69 | — | 1.96±0.01 | — |
| β-Ocimene | 10.22 | — | — | 2.23±0 |
| β-elemene | 18.45 | — | — | 1.95±0 |
| Copaene | 18.08 | — | — | 1.81±0 |
| Cyclohexane, 1-ethenyl-1-methyl-2-(1-methylethenyl)-4-(1-methylethylidene)- | 17.16 | — | — | 0.96±0 |
| α-Cubebene | 18.52 | — | — | 0.61±0 |
| Ylangene | 17.98 | — | — | 0.32±0 |
| Bicyclo[5.3.0]decane, 2-methylene-5-(1-methylvinyl)-8-methyl- | 16.97 | — | — | 0.25±0 |
| β-Vatirenene | 25.47 | — | — | 0.03±0 |
| 10-Heneicosene (c,t) | 33.54 | — | — | 0.02±0 |
| 17-Pentatriacontene | 30.63 | — | — | 0.01±0 |
| **Others** |  |  |  |  |
| Cyclohexane, 2-ethenyl-1,1-dimethyl-3-methylene- | 11.89 | 6.66±0.05 | — | — |
| Tricyclo[3.1.0.0(2,4)]hexane, 3,6-diethyl-3,6-dimethyl-, trans- | 23.06 | — | — | 4.09±0 |
| Ledene oxide-(II) | 22.40 | — | — | 0.19±0 |
| 4-Pentenal, 2-methyl- | 4.48 | 10.93±0.03 | 5.84±0.02 | — |
| Benzaldehyde | 8.23 | — | — | 0.40±0 |
| 2-Cyclopenten-1-one, 3-methyl-2-(2-pentenyl)-, (Z)- | 18.51 | — | 1.46±0 | — |
| 1H-Inden-1-one, 2,3-dihydro-3,3,4,6-tetramethyl- | 26.13 | — | — | 0.44±0 |
| p-Xylene | 6.22 | — | — | 0.83±0 |
| Indole | 16.40 | — | — | 5.02±0 |
| Benzenepropanoic acid, α-(hydroxyimino)- | 12.58 | — | — | 0.09±0 |

**Table S16. The candidate genes involved in the benzenoid/phenylpropanoid synthesis pathways in *J. sambac* cultivar JSDB.**

| Pathway | Gene name | Gene ID | Location |
| --- | --- | --- | --- |
| Shikima pathway | DAHPS | contig34.g31 | contig34:476892-479757(-) |
|  | DAHPS | contig5933.g2 | contig5933:7519-8003(-) |
|  | DAHPS | contig250.g16 | contig250:167958-171930(-) |
|  | DAHPS | contig1035.g6 | contig1035:54261-57142(-) |
|  | DAHPS | contig5933.g1 | contig5933:5210-7492(-) |
|  | DHQS | contig806.g13 | contig806:97279-112088(+) |
|  | DHQS | contig806.g14 | contig806:114101-127102(+) |
|  | DHQS | contig806.g15 | contig806:132528-135702(+) |
|  | DHQS | contig1561.g10 | contig1561:77064-80209(+) |
|  | DHD/SDH | contig2011.g3 | contig2011:11413-16105(+) |
|  | DHD/SDH | contig254.g38 | contig254:240745-244408(-) |
|  | SK | contig343.g13 | contig343:196216-201188(+) |
|  | SK | contig103.g42 | contig103:372875-376739(-) |
|  | SK | contig1417.g7 | contig1417:81556-86216(+) |
|  | EPSPS | contig1886.g2 | contig1886:19557-22506(+) |
|  | CS | contig104.g42 | contig104:384020-392623(-) |
| Phenylpyruvate&Arogenate pathway | CM | contig5358.g1 | contig5358:6605-8538(-) |
|  | CM | contig5399.g1 | contig5399:922-2568(+) |
|  | CM | contig1191.g3 | contig1191:11672-13312(+) |
|  | CM | contig2189.g7 | contig2189:64208-66583(+) |
|  | CM | contig2777.g1 | contig2777:820-3194(+) |
|  | CM | contig40.g59 | contig40:523583-526662(-) |
|  | PPA-AT | contig4477.g1 | contig4477:12297-14878(+) |
|  | PPA-AT | contig3864.g3 | contig3864:16963-21071(-) |
|  | ADT/PDT | contig97.g45 | contig97:382764-387209(-) |
|  | ADT/PDT | contig1765.g5 | contig1765:78762-80042(-) |
|  | ADT/PDT | contig338.g3 | contig338:34795-35404(-) |
|  | ADT/PDT | contig42.g35 | contig42:381730-388678(+) |
|  | ADT/PDT | contig427.g1 | contig427:68374-69672(-) |
| benzenoid/ phenylpropanoid biosynthesis | PAL | contig150.g3 | contig150:75595-79277(+) |
|  | PAL | contig150.g5 | contig150:85615-88584(+) |
|  | PAL | contig150.g6 | contig150:94334-97966(+) |
|  | PAL | contig2031.g1 | contig2031:467-855(-) |
|  | PAL | contig2031.g2 | contig2031:3613-6994(-) |
|  | PAL | contig5310.g1 | contig5310:1861-5119(-) |
|  | PAL | contig5310.g2 | contig5310:12197-13676(-) |
|  | PAL | contig1523.g8 | contig1523:65955-69329(+) |
|  | BEAT | contig1225.g6 | contig1225:80993-82276(+) |
|  | BEAT | contig1225.g7 | contig1225:97837-99117(+) |
|  | BEAT | contig913.g1 | contig913:27507-28571(+) |
|  | BEAT | contig5009.g2 | contig5009:15148-16437(-) |
|  | BEAT | contig234.g10 | contig234:69103-70425(+) |
|  | BEAT | contig3937.g1 | contig3937:3626-4946(+) |
|  | BEAT | contig2158.g3 | contig2158:32941-34214(-) |
|  | BEAT | contig250.g19 | contig250:195631-196362(-) |
|  | BEAT | contig4692.g1 | contig4692:21543-22868(+) |
|  | BEAT | contig4577.g1 | contig4577:22062-23372(+) |
|  | BEAT | contig898.g4 | contig898:44907-45707(+) |
|  | BEAT | contig898.g5 | contig898:51108-51491(+) |
|  | BEAT | contig898.g6 | contig898:54752-55582(+) |
|  | BEAT | contig898.g7 | contig898:55746-56090(+) |
|  | BEAT | contig422.g2 | contig422:25958-27395(+) |
|  | BEAT | contig422.g1 | contig422:17402-17986(-) |
|  | BEAT | contig441.g17 | contig441:196746-198017(-) |
|  | BEAT | contig441.g16 | contig441:194318-196233(+) |
|  | BEAT | contig6.g16 | contig6:126322-128192(-) |
|  | AAO4 | contig2194.g8 | contig2194:62655-68052(-) |
|  | AAO4 | contig4089.g1 | contig4089:450-2204(-) |
|  | AAO4 | contig700.g9 | contig700:96285-100612(+) |
|  | AAO4 | contig958.g9 | contig958:124704-129909(+) |
|  | SAMT | contig1566.g7 | contig1566:50988-55858(-) |
|  | SAMT | contig995.g4 | contig995:55258-58543(+) |
|  | SAMT | contig965.g8 | contig965:55050-56828(+) |
|  | BAMT | contig965.g8 | contig965:55050-56828(+) |
|  | BAMT | contig197.g22 | contig197:166417-169516(-) |

Note: The shading marked in red and yellow represent WGD and tandem duplication event, respectively. The full names of enzymes were listed in the Figure 3.

**Table S17. Reading mapping summary of *J. sambac* cultivar JSDB at different developmental stages.**

| Sample | Total raw reads | Total clean reads | Total clean data (bp) | Clean reads ratio (%) | Clean data ratio (%) |
| --- | --- | --- | --- | --- | --- |
| S1-1 | 40,825,112 | 37,832,854 | 5,712,760,954 | 92.67 | 92.67 |
| S1-2 | 40,417,498 | 37,480,060 | 5,659,489,060 | 92.73 | 92.73 |
| S1-3 | 40,015,414 | 37,054,862 | 5,595,284,162 | 92.60 | 92.60 |
| S2-1 | 43,914,024 | 40,644,494 | 6,137,318,594 | 92.55 | 92.55 |
| S2-2 | 40,528,168 | 37,519,934 | 5,665,510,034 | 92.57 | 92.57 |
| S2-3 | 43,128,504 | 39,890,370 | 6,023,445,870 | 92.49 | 92.49 |
| S3-1 | 43,648,262 | 40,407,196 | 6,101,486,596 | 92.57 | 92.57 |
| S3-2 | 41,244,892 | 38,114,794 | 5,755,333,894 | 92.41 | 92.41 |
| S3-3 | 42,386,262 | 39,243,528 | 5,925,772,728 | 92.58 | 92.58 |

**Table S18. The candidate genes involved in the terpenoid synthesis pathways in *J. sambac* cultivar JSDB.**

| Pathway | Gene name | Gene ID | Location |
| --- | --- | --- | --- |
| MVA pathway | ACAT | contig13.g7 | contig13:199583-204884(+) |
|  | ACAT | contig561.g13 | contig561:96236-101322(-) |
|  | HMGS | contig3224.g1 | contig3224:4331-8800(+) |
|  | HMGR | contig1594.g1 | contig1594:148-1485(+) |
|  | HMGR | contig549.g16 | contig549:132854-134173(-) |
|  | HMGR | contig2431.g2 | contig2431:16308-19272(-) |
|  | HMGR | contig624.g16 | contig624:128460-130208(-) |
|  | HMGR | contig137.g29 | contig137:317093-321088(+) |
|  | MVK | contig1184.g16 | contig1184:112288-114704(+) |
|  | PMK | contig146.g15 | contig146:297100-316137(+) |
|  | MDC | contig505.g25 | contig505:192213-195326(-) |
|  | IDI | contig134.g17 | contig134:313657-317236(+) |
|  | FPPS | contig428.g21 | contig428:168502-170578(+) |
| MEP pathway | DXS | contig254.g35 | contig254:232154-236086(+) |
|  | DXS | contig1719.g3 | contig1719:12154-14154(-) |
|  | DXS | contig189.g8 | contig189:110764-113614(-) |
|  | DXR | contig352.g11 | contig352:60543-63983(+) |
|  | MCT | contig389.g22 | contig389:188669-191871(-) |
|  | CMK | contig1788.g11 | contig1788:66361-69266(-) |
|  | MCS | contig164.g5 | contig164:131283-133200(+) |
|  | MCS | contig164.g7 | contig164:152195-152422(+) |
|  | HDS | contig2022.g3 | contig2022:9105-13691(-) |
|  | HDR | contig2112.g2 | contig2112:12465-15790(+) |
|  | HDR | contig577.g23 | contig577:125325-128778(+) |
|  | GPPS | contig990.g19 | contig990:110611-111516(-) |
|  | GGPPS | contig461.g8 | contig461:61603-64714(-) |
|  | GGPPS | contig3396.g1 | contig3396:7370-16873(-) |
|  | GGPPS | contig4356.g2 | contig4356:20000-21097(-) |
|  | GGPPS | contig3659.g1 | contig3659:13090-14172(-) |
|  | GGPPS | contig69.g4 | contig69:235200-236628(+) |
|  | GGPPS | contig69.g5 | contig69:243422-244006(+) |
|  | GGPPS | contig1105.g9 | contig1105:113957-115259(-) |

Note: The shading marked in red represent WGD event. The full names of enzymes were listed in the Figure 4.

**Table S19.** **The *cis*-element number of *TPSs* promoter in *J. sambac* cultivar JSDB.**

| Gene ID | Defense and stress responsive | | | | | |  | Phytohormone responsive | | | | | | |  | Plant growth and development | | |
| --- | --- | --- | --- | --- | --- | --- | --- | --- | --- | --- | --- | --- | --- | --- | --- | --- | --- | --- |
|  | MYB bind-element | bHLH bind-element | WRKY bind-element | LTR | TC-rich repeats | ARE |  | P-box | TCA-element | ABRE | TGA-element | CGTCA-motif | TGACG-motif | GARE-motif |  | CAT-box | GCN4-motif | O_2_-site |
| contig88.g2 | 8 | 2 | 1 | 0 | 2 | 3 |  | 0 | 0 | 1 | 0 | 4 | 4 | 0 |  | 0 | 1 | 1 |
| contig156.g34 | 6 | 8 | 2 | 0 | 0 | 1 |  | 0 | 1 | 6 | 2 | 5 | 5 | 0 |  | 0 | 0 | 1 |
| contig203.g7 | 8 | 4 | 2 | 0 | 1 | 2 |  | 0 | 1 | 4 | 1 | 3 | 3 | 0 |  | 0 | 0 | 2 |
| contig203.g8 | 5 | 10 | 2 | 1 | 0 | 4 |  | 0 | 0 | 6 | 0 | 2 | 2 | 1 |  | 2 | 0 | 0 |
| contig304.g22 | 15 | 12 | 0 | 1 | 0 | 4 |  | 1 | 1 | 10 | 1 | 6 | 6 | 0 |  | 1 | 0 | 0 |
| contig304.g23 | 10 | 5 | 2 | 2 | 1 | 3 |  | 0 | 0 | 5 | 0 | 4 | 4 | 1 |  | 0 | 0 | 0 |
| contig386.g6 | 4 | 6 | 1 | 5 | 0 | 1 |  | 1 | 2 | 4 | 0 | 4 | 4 | 0 |  | 0 | 0 | 1 |
| contig386.g7 | 10 | 2 | 4 | 2 | 0 | 5 |  | 0 | 1 | 5 | 1 | 3 | 3 | 0 |  | 0 | 0 | 0 |
| contig406.g13 | 7 | 3 | 1 | 1 | 1 | 1 |  | 0 | 1 | 3 | 1 | 1 | 1 | 0 |  | 1 | 0 | 0 |
| contig406.g15 | 13 | 5 | 4 | 0 | 0 | 0 |  | 0 | 0 | 4 | 3 | 1 | 1 | 0 |  | 0 | 0 | 0 |
| contig406.g17 | 0 | 6 | 0 | 3 | 0 | 8 |  | 0 | 1 | 5 | 1 | 2 | 2 | 0 |  | 0 | 0 | 0 |
| contig406.g18 | 9 | 2 | 0 | 0 | 2 | 3 |  | 0 | 0 | 3 | 0 | 1 | 1 | 0 |  | 0 | 1 | 1 |
| contig445.g1 | 6 | 4 | 5 | 0 | 0 | 3 |  | 0 | 0 | 4 | 0 | 2 | 2 | 0 |  | 2 | 1 | 0 |
| contig524.g5 | 11 | 3 | 0 | 2 | 2 | 7 |  | 0 | 0 | 3 | 0 | 1 | 1 | 0 |  | 2 | 2 | 0 |
| contig767.g5 | 7 | 10 | 0 | 0 | 0 | 0 |  | 0 | 1 | 8 | 1 | 1 | 1 | 0 |  | 2 | 0 | 0 |
| contig854.g11 | 11 | 6 | 2 | 1 | 1 | 0 |  | 1 | 0 | 6 | 0 | 3 | 3 | 1 |  | 2 | 0 | 0 |
| contig1096.g3 | 6 | 1 | 2 | 2 | 2 | 6 |  | 2 | 0 | 0 | 1 | 2 | 2 | 0 |  | 1 | 0 | 0 |
| contig1588.g3 | 6 | 2 | 3 | 0 | 0 | 1 |  | 0 | 1 | 2 | 0 | 3 | 3 | 0 |  | 0 | 0 | 0 |
| contig1673.g4 | 1 | 6 | 0 | 0 | 1 | 2 |  | 0 | 1 | 6 | 1 | 5 | 5 | 0 |  | 0 | 0 | 0 |
| contig1673.g5 | 8 | 5 | 0 | 1 | 0 | 6 |  | 1 | 1 | 3 | 1 | 5 | 5 | 1 |  | 0 | 0 | 2 |
| contig1768.g1 | 10 | 2 | 1 | 0 | 0 | 1 |  | 0 | 2 | 3 | 0 | 2 | 2 | 1 |  | 1 | 2 | 1 |
| contig1768.g2 | 17 | 2 | 4 | 1 | 0 | 2 |  | 0 | 1 | 3 | 2 | 2 | 2 | 2 |  | 0 | 0 | 1 |
| contig1768.g4 | 4 | 2 | 4 | 0 | 0 | 10 |  | 0 | 1 | 1 | 1 | 3 | 3 | 0 |  | 0 | 0 | 2 |
| contig2037.g2 | 10 | 4 | 2 | 0 | 1 | 6 |  | 0 | 1 | 4 | 0 | 3 | 3 | 0 |  | 1 | 1 | 1 |
| contig2118.g7 | 12 | 6 | 1 | 0 | 1 | 4 |  | 0 | 1 | 4 | 0 | 4 | 4 | 0 |  | 0 | 0 | 1 |
| contig2155.g2 | 9 | 2 | 1 | 2 | 0 | 4 |  | 0 | 0 | 2 | 2 | 4 | 4 | 0 |  | 0 | 0 | 2 |
| contig3121.g3 | 10 | 0 | 0 | 0 | 0 | 0 |  | 0 | 0 | 0 | 0 | 0 | 0 | 0 |  | 0 | 0 | 0 |
| contig3121.g5 | 2 | 3 | 2 | 0 | 0 | 1 |  | 0 | 2 | 1 | 0 | 1 | 1 | 0 |  | 0 | 1 | 1 |
| contig3306.g4 | 5 | 4 | 2 | 1 | 1 | 1 |  | 0 | 0 | 4 | 0 | 2 | 2 | 0 |  | 0 | 0 | 1 |
| contig3371.g1 | 9 | 0 | 2 | 0 | 0 | 1 |  | 0 | 1 | 1 | 1 | 2 | 2 | 0 |  | 0 | 0 | 0 |
| contig4421.g1 | 6 | 2 | 1 | 0 | 2 | 4 |  | 0 | 0 | 1 | 0 | 4 | 4 | 0 |  | 0 | 1 | 1 |

The shading marked in yellow represent tandem duplication event.
